# Body roundness index and its role in predicting COPD risk: insights from the English Longitudinal Study of Aging and the health and retirement study

**DOI:** 10.3389/fmed.2025.1670309

**Published:** 2025-10-16

**Authors:** Longqian Li, ZhuoLin Qin, Mingzhi Lin, Chun zhang, Cheng Wang

**Affiliations:** ^1^The Second Clinical Medical College, Lanzhou University, Lanzhou, China; ^2^Affiliated Hospital of Jiangsu University, Zhenjiang, China; ^3^Department of Thoracic Surgery, Lanzhou University Second Hospital, Lanzhou, China

**Keywords:** Chronic obstructive pulmonary disease, Body roundness index, the English Longitudinal Study of Aging, the Health and Retirement Study, restricted cubic spline

## Abstract

**Background:**

Chronic obstructive pulmonary disease (COPD) is one of the most common chronic respiratory diseases worldwide. This study aims to investigate the relationship between the Body Roundness Index (BRI) and COPD in individuals aged 45 and older.

**Methods:**

This study included 5818 participants from waves 2 to 9 (2004–2019) of the English Longitudinal Study of Aging (ELSA) and 6928 participants from waves 8 to 10 (2006–2021) of the Health and Retirement Study (HRS). Initially, univariate analysis, univariate Cox regression analysis, and trend analysis were conducted to preliminarily screen the variables. The variance inflation factor (VIF) was used to detect multicollinearity and ensure the independence of the selected variables. Subsequently, multivariate logistic regression and multivariate Cox regression models were employed to assess the relationship between the Body Roundness Index (BRI) and chronic obstructive pulmonary disease (COPD). Restricted cubic spline (RCS) analysis was applied to further explore the nonlinear relationship between BRI and COPD. Finally, sensitivity analysis was performed to validate the robustness of the model results.

**Results:**

The results from both datasets indicate a significant association between the Body Roundness Index (BRI) and chronic obstructive pulmonary disease (COPD) (ELSA: OR (95% CI) = 1.193 (1.074–1.321), *P* = 0.001; HRS: OR (95% CI) = 1.160 (1.094–1.228), *P* < 0.001). As BRI increases, the incidence of newly diagnosed COPD significantly rises (ELSA: HR (95% CI) = 1.149 (1.034–1.273), *P* = 0.009; HRS: HR (95% CI) = 1.114 (1.054–1.177), *P* < 0.001). The optimal cutoff analysis revealed a significant difference in COPD risk between the high and low BRI groups (ELSA: *P* = 0.0037; HRS: *P* = 0.0085). Restricted cubic spline (RCS) analysis further demonstrated a “J-shaped” relationship between BRI and COPD.

**Conclusion:**

This study demonstrates a significant association between the Body Roundness Index (BRI) and chronic obstructive pulmonary disease (COPD). The increase in BRI is significantly associated with both the incidence of COPD and newly diagnosed cases. Restricted cubic spline (RCS) analysis further reveals a “J-shaped” relationship between BRI and COPD, suggesting that BRI may serve as a potential predictive tool for COPD risk.

## 1 Introduction

Chronic obstructive pulmonary disease (COPD) is a chronic respiratory disease characterized by airflow limitation and persistent respiratory symptoms, often accompanied by airway inflammation and lung tissue damage (1). In 2021, the global prevalence of COPD was 360 million, resulting in 5.9 million deaths, making it the fourth leading cause of death worldwide (2). Despite the implementation of management and preventive strategies, the global burden of COPD remains significant. According to predictions by the World Health Organization, by 2060, COPD will become the fourth leading cause of death globally, with an estimated annual death toll exceeding 5.4 million (3–5). In particular, the dual burden of COPD and lower respiratory tract infections (LRI) in low-income regions exacerbates the impact of the disease, posing substantial challenges to public health systems (2).

The occurrence of COPD is closely associated with several factors, particularly smoking, gender, and age (6, 7). Studies have shown that the incidence of COPD increases significantly with age, especially in individuals over 45 years old (6, 8). Additionally, obesity, particularly the accumulation of abdominal fat, has been shown to be strongly associated with the development of COPD (9–12). A large body of research indicates a positive correlation between waist circumference, fat accumulation, and COPD risk (4). Lipid accumulation product (LAP), a key indicator for evaluating abdominal fat, has been significantly linked to COPD risk (13). Studies have found that an increase in LAP values correlates significantly with the risk of COPD, especially in individuals with greater abdominal fat accumulation, where the risk of developing COPD is notably higher (4, 13). However, LAP primarily focuses on waist circumference and does not fully account for other aspects of fat distribution, limiting its application in COPD risk prediction.

Although BMI and LAP offer certain advantages in assessing abdominal fat, they do not fully consider the specific physiological mechanisms of fat distribution (14). Particularly, visceral fat is not just a fat store but also exerts profound impacts on health through a series of complex metabolic and biological pathways (15, 16). Research has shown that excessive accumulation of visceral fat is closely associated with airway inflammation, oxidative stress, and changes in respiratory mechanics–factors that are all critically involved in the development and progression of COPD (17, 18). BRI, by combining height, weight, and waist circumference, provides a more precise representation of abdominal fat accumulation, particularly the distribution of visceral fat (19–21). As such, BRI exhibits a stronger predictive ability than BMI and LAP in revealing the negative impact of abdominal fat on lung function (22). Compared to traditional measurement indices, BRI more sensitively captures the effects of visceral fat, such as triggering inflammatory responses, exacerbating oxidative stress, and altering respiratory mechanics, all of which contribute to COPD (23, 24). Through these mechanisms, BRI offers important predictive value for the early diagnosis and intervention of COPD.

In this context, the aim of our study is to investigate the relationship between changes in the Body Roundness Index (BRI) and both the risk of developing and the prevalence of COPD, further exploring the potential link between them. Using data from the Health and Retirement Study (HRS) and the English Longitudinal Study of Aging (ELSA), we aim to comprehensively examine this association across diverse populations. The focus of our study is to assess the relationship between BRI and the risk of both developing new COPD cases and the prevalence of existing COPD, hypothesizing that an increase in BRI may elevate the risk of both the onset and the prevalence of COPD.

## 2 Materials and methods

### 2.1 Study design and sample

All data are from two main longitudinal datasets: the English Longitudinal Study on Aging (ELSA) and the Health and Retirement Study (HRS) from the US (25, 26). All ELSA and HRS surveys and follow-ups were approved by the Multi-Center Research Ethics Service and Institute for Social Research and Survey Research Center of the University of Michigan, respectively. All the participants provided informed consent.

**FIGURE 1:**
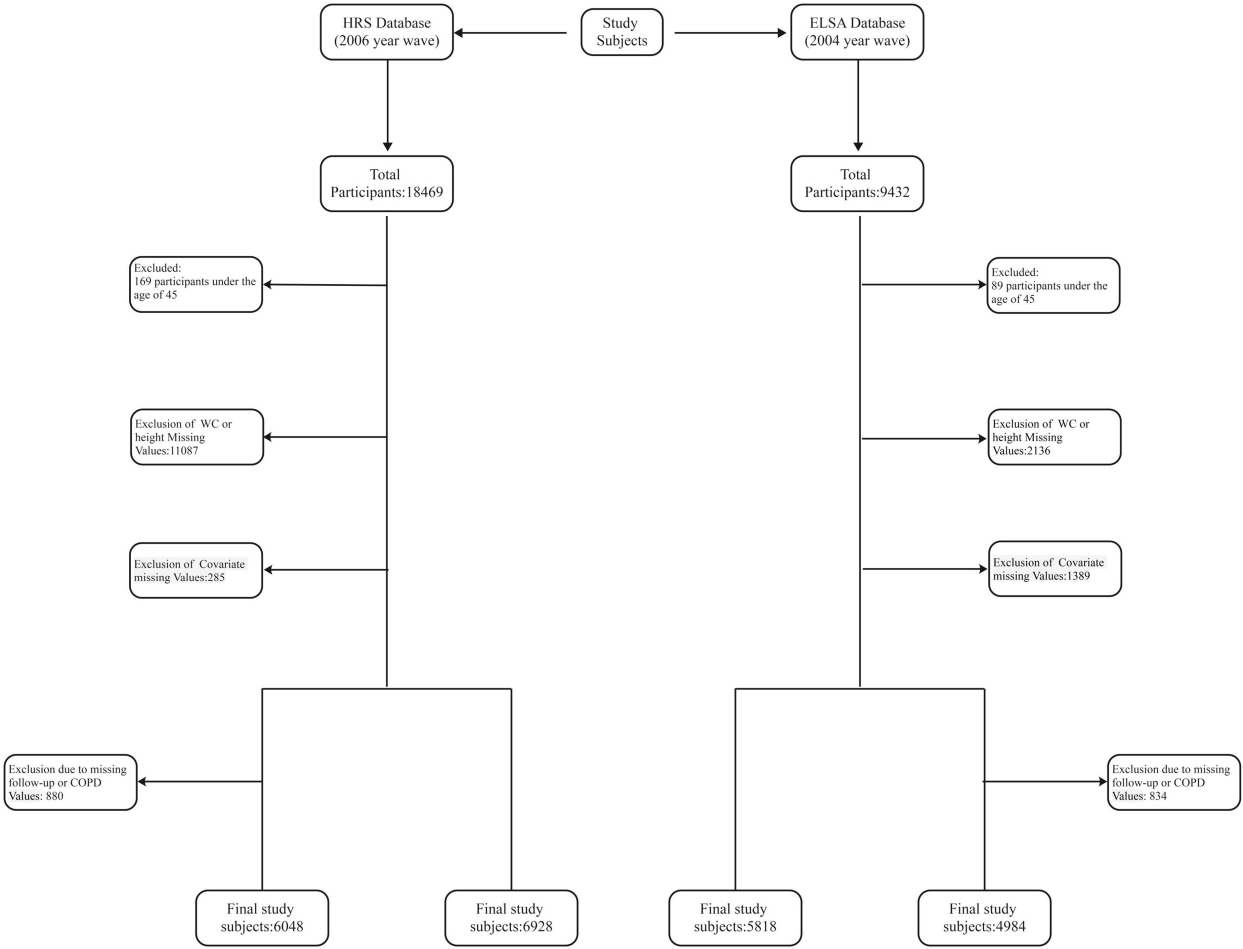
Flowchart of the selection of the study population. HRS indicates the Health and Retirement Study. ELSA indicates the English Longitudinal Study on Aging.

We extracted data from participants in the second to ninth waves of the English Longitudinal Study of Aging (ELSA) (2004–2019) and the eighth to tenth waves of the Health and Retirement Study (HRS) (2006–2021), with the second wave of ELSA and the eighth wave of HRS serving as baseline data. Based on strict exclusion criteria, we selected participants who met the following conditions: (1) age <45 years; (2) missing waist circumference or height data; (3) missing covariate information; (4) no Chronic Obstructive Pulmonary Disease (COPD) information during follow-up. The selection process is depicted in Figure 1.

In the HRS, a total of 6928 participants were included in the cross-sectional baseline analysis, and 6048 participants were included in the longitudinal Cox regression analysis. In the ELSA, a total of 5818 participants were included in the cross-sectional baseline analysis, and 4984 participants were included in the longitudinal Cox regression analysis

### 2.2 Assessment of COPD

The diagnosis of COPD was based on self-report, where participants were asked, “Has a doctor ever told you that you have chronic lung disease, such as chronic bronchitis or emphysema?” (27–29).

### 2.3 Assessment of BRI

The BRI is an indicator used to evaluate body shape and quantify the accumulation of abdominal fat. Unlike traditional body mass index (BMI), BRI more accurately reflects the distribution of abdominal fat, especially the accumulation of visceral fat. The calculation formula for BRI is as follows (30):


$$\mathrm{BRI}=364.2-365.5\,\sqrt{1-{\left(\frac{\mathrm{WC}\,\left(m\right)÷2\,\pi }{0.5\,\mathrm{height}\,\left(m\right)}\right)}^{2}}$$

### 2.4 Covariates

In this study, we included various potential covariates that may be associated with the incidence of Chronic Obstructive Pulmonary Disease (COPD) to ensure the comprehensiveness and accuracy of the analysis. The specific covariates include: age, sex, race, education level, marital status, alcohol consumption, smoking status, Smoking quantity(per day), hypertension, diabetes mellitus (DM), cancer, COPD, Emotional/neurological/mental issues, arthritis, cardiovascular disease (CVD), vigorous physical activity, moderate physical activity, light physical activity, CES-D (Center for Epidemiologic Studies Depression Scale), and body mass index (BMI) (31).

Education level was categorized into three groups: less than high school, high school and vocational training, and higher education. Race was categorized as White and Non-white. Cardiovascular disease (CVD) included heart conditions, stroke, myocardial infarction (including myocardial infarction or coronary thrombosis), angina, congestive heart failure, heart murmurs, past reports of heart attacks or myocardial infarction within the last 2 years, and arrhythmias.

The frequency of vigorous physical activity was assessed by the question from the physician: “How often do you participate in the following activities: vigorous exercise such as running or jogging, swimming, cycling, aerobics or gym workouts, tennis, or digging with a shovel or spade?” Response options included: more than once a week, once a week, 1–3 times a month, or almost never. The frequency of moderate physical activity was similarly assessed: “How often do you participate in moderate-intensity activities, such as gardening, car washing, walking at a moderate pace, dancing, floor cleaning, or stretching exercises?” Response options included: more than once a week, once a week, 1–3 times a month, or almost never. The frequency of light physical activity was assessed with the question: “How often do you participate in light-intensity activities, such as vacuuming, doing laundry, or home repairs?” Response options included: more than once a week, once a week, 1–3 times a month, or almost never.

The Center for Epidemiologic Studies Depression Scale (CES-D) includes three negative affect domains, two positive affect domains, and three somatic symptom domains. The total score ranges from 0 to 8, with a cutoff score of 3 to categorize participants into high and low symptom levels. The negative affective domain includes items such as “feeling down”, “feeling lonely” or “feeling sad”, while somatic symptoms include items like “everything takes effort”, “troubled sleep” and “unable to get started” (31–34).

### 2.5 Statistical analyses

This study utilized various statistical methods for a comprehensive analysis of the data. Descriptive statistical analysis was first performed on the baseline demographic characteristics of ELSA wave 2 and HRS wave 8. Continuous variables were expressed as mean ± standard deviation, and intergroup comparisons were conducted using the *T*-test or Kruskal-Wallis rank-sum test. Categorical variables were presented as percentages, with comparisons made using the chi-square test. Univariate Cox regression analysis was employed to assess the relationship with incident COPD. The BRI and 95% confidence intervals (CI) for each cycle of HRS and ELSA were calculated, and comparisons were made for both overall and subgroup analyses within each cycle. Trends were assessed using Mann-Kendall tests and linear regression.

To ensure the stability of the models and minimize the risk of multicollinearity, variance inflation factor (VIF) was used for the selection of independent variables. VIF quantifies the level of collinearity between variables, and variables with a VIF greater than 5 were excluded to enhance the model’s stability and interpretability ([22]). Multivariable logistic regression analysis was performed to compute the odds ratio (OR) and its 95% confidence interval (CI) to evaluate the association between BRI and COPD. Cox regression models further explored the hazard ratio (HR) between BRI and incident COPD. Additionally, restricted cubic spline (RCS) analysis was applied to capture potential nonlinear relationships between BRI and COPD. Sensitivity analyses were conducted in two stages: first, by excluding outliers with BRI values below the 5th percentile and above the 95th percentile, and second, by performing multiple imputation to handle missing data. These two approaches were used to assess the robustness and consistency of the results. Finally, subgroup analyses were conducted to further explore the relationship between BRI and COPD.

All statistical analyses were performed using R software (version 4.4.2).

## 3 Results

### 3.1 Baseline characteristics of participants

A total of 12,746 participants (Table 1) were included in this study (HRS: 6,928; ELSA: 5,818). Among the ELSA participants, 343 (5.8%) were diagnosed with COPD, with an average age of 68.3 years, which was significantly higher than the normal group (65.4 years, *P*<0.001). The COPD group also had a significantly higher average daily smoking rate (2.8 cigarettes) compared to the normal group (1.4 cigarettes, *P*<0.001). There were significant differences between the two groups in terms of education level, marital status, alcohol consumption, smoking, hypertension, cardiovascular disease (CVD), CESD scores, and BMI (*P*<0.05). Notably, the COPD group had a higher proportion of individuals with higher education (23.6% with university education), alcohol consumption (84%), smoking (27.4%), CVD (32.4%), and CESD scores above 2, compared to the normal group.

**TABLE 1:** Population baseline characteristics.

Characteristic	Elsa			HRS		
	Non-COPD	COPD	P-Value	Non-COPD	COPD	P-Value
N	5475	343		6349	579	
Age	65.4 ± 9.1	68.3 ± 9.0	<0.001	67.4 ± 10.3	69.2 ± 9.6	<0.001
Smoking quantity(per day)	1.4 ± 5.1	2.8 ± 6.4	<0.001	1.9 ± 6.5	5.0 ± 10.0	<0.001
BRI	5.0 ± 1.6	5.3 ± 1.7	0.004	5.6 ± 2.0	6.1 ± 2.5	<0.001
Sex			0.112			0.320
Female	2923 (53.4%)	168 (49.0%)		3710 (58.4%)	326 (56.3%)	
Male	2552 (46.6%)	175 (51.0%)		2639 (41.6%)	253 (43.7%)	
Race			0.795			0.011
White	62 (1.1%)	4 (1.2%)		1131 (17.8%)	79 (13.6%)	
Other	5413 (98.9%)	339 (98.8%)		5218 (82.2%)	500 (86.4%)	
Education			<0.001			<0.001
Less than high school	2065 (37.7%)	183 (53.4%)		1141 (18.0%)	154 (26.6%)	
High school	2600 (47.5%)	137 (39.9%)		3753 (59.1%)	354 (61.1%)	
Higher	810 (14.8%)	23 (6.7%)		1455 (22.9%)	71 (12.3%)	
Marital			<0.001			<0.001
Never married	255 (4.7%)	21 (6.1%)		204 (3.2%)	17 (2.9%)	
Married	3859 (70.5%)	201 (58.6%)		4197 (66.1%)	330 (57.0%)	
Other	1361 (24.9%)	121 (35.3%)		1948 (30.7%)	232 (40.1%)	
Alcohol			<0.001			0.011
No	516 (9.4%)	55 (16.0%)		2983 (47.0%)	304 (52.5%)	
Yes	4959 (90.6%)	288 (84.0%)		3366 (53.0%)	275 (47.5%)	
Smoke			<0.001			<0.001
No	4759 (86.9%)	249 (72.6%)		5569 (87.7%)	414 (71.5%)	
Yes	716 (13.1%)	94 (27.4%)		780 (12.3%)	165 (28.5%)	
Hypertension			0.036			<0.001
No	3329 (60.8%)	189 (55.1%)		3035 (47.8%)	231 (39.9%)	
Yes	2146 (39.2%)	154 (44.9%)		3314 (52.2%)	348 (60.1%)	
Diabetes mellitus			0.880			0.006
No	5072 (92.6%)	317 (92.4%)		5235 (82.5%)	451 (77.9%)	
Yes	403 (7.4%)	26 (7.6%)		1114 (17.5%)	128 (22.1%)	
Cancer			0.390			<0.001
No	5095 (93.1%)	315 (91.8%)		5506 (86.7%)	444 (76.7%)	
Yes	380 (6.9%)	28 (8.2%)		843 (13.3%)	135 (23.3%)	
Emotional/neurological/mental issues			0.544			<0.001
No	5046 (92.2%)	313 (91.3%)		5555 (87.5%)	417 (72.0%)	
Yes	429 (7.8%)	30 (8.7%)		794 (12.5%)	162 (28.0%)	
CVD			<0.001			<0.001
No	4314 (78.8%)	232 (67.6%)		4810 (75.8%)	328 (56.6%)	
Yes	1161 (21.2%)	111 (32.4%)		1539 (24.2%)	251 (43.4%)	
Vigorous activities			<0.001			<0.001
Never	3120 (57.0%)	266 (77.6%)		3806 (59.9%)	449 (77.5%)	
1–3 times per month	640 (11.7%)	23 (6.7%)		444 (7.0%)	26 (4.5%)	
Once a week	599 (10.9%)	25 (7.3%)		538 (8.5%)	27 (4.7%)	
At least once a week	1116 (20.4%)	29 (8.5%)		1372 (21.6%)	60 (10.4%)	
Every day				189 (3.0%)	17 (2.9%)	
Moderate activity			<0.001			<0.001
Never	682 (12.5%)	100 (29.2%)		1102 (17.4%)	183 (31.6%)	
1–3 times per month	403 (7.4%)	37 (10.8%)		519 (8.2%)	61 (10.5%)	
Once a week	834 (15.2%)	48 (14.0%)		979 (15.4%)	82 (14.2%)	
At least once a week	3556 (64.9%)	158 (46.1%)		3045 (48.0%)	200 (34.5%)	
Every day				704 (11.1%)	53 (9.2%)	
Mild activity			<0.001			<0.001
Never	334 (6.1%)	51 (14.9%)		442 (7.0%)	98 (16.9%)	
1–3 times per month	189 (3.5%)	16 (4.7%)		394 (6.2%)	35 (6.0%)	
Once a week	542 (9.9%)	29 (8.5%)		1382 (21.8%)	140 (24.2%)	
At least once a week	4410 (80.5%)	247 (72.0%)		3320 (52.3%)	254 (43.9%)	
Every day				811 (12.8%)	52 (9.0%)	
CESD			<0.001			<0.001
0	2334 (42.6%)	79 (23.0%)		3056 (48.1%)	169 (29.2%)	
1	1458 (26.6%)	84 (24.5%)		1352 (21.3%)	124 (21.4%)	
2	611 (11.2%)	52 (15.2%)		704 (11.1%)	91 (15.7%)	
3	387 (7.1%)	38 (11.1%)		421 (6.6%)	59 (10.2%)	
4	234 (4.3%)	27 (7.9%)		247 (3.9%)	29 (5.0%)	
5	150 (2.7%)	26 (7.6%)		207 (3.3%)	36 (6.2%)	
6	152 (2.8%)	16 (4.7%)		178 (2.8%)	33 (5.7%)	
7	93 (1.7%)	13 (3.8%)		117 (1.8%)	21 (3.6%)	
8	56 (1.0%)	8 (2.3%)		67 (1.1%)	17 (2.9%)	
BMI			0.009			<0.001
Underweight	35 (0.6%)	6 (1.7%)		49 (0.8%)	14 (2.4%)	
Normal	1476 (27.0%)	110 (32.1%)		1487 (23.4%)	163 (28.2%)	
Overweight	2397 (43.8%)	129 (37.6%)		2377 (37.4%)	175 (30.2%)	
Obesity	1567 (28.6%)	98 (28.6%)		2436 (38.4%)	227 (39.2%)	

CVD, cardiovascular disease; BRI, body roundness index; CESD, the Center for Epidemiologic Studies Depression Scale (CES-D).

In the HRS dataset, 579 participants (8.4%) were diagnosed with COPD, with an average age of 69.2 years, which was significantly higher than the normal group (*P*<0.001). The average daily smoking rate was also significantly higher in the COPD group (5.0 cigarettes) compared to the normal group (*P*<0.001). Interestingly, in both datasets, the proportion of overweight individuals was higher in the normal group than in the COPD group, and the proportion of individuals with a normal BMI was lower in the COPD group

### 3.2 Temporal trends of BRI

The time trend of BRI is shown in Supplementary Table 1. In the HRS data, the average BRI increased from 5.642 (95% CI, 5.594–5.691) to 6.224 (95% CI, 6.102–6.346). Using the average BRI from the 2006–2007 cycle as a reference, the change range was 0.162–0.582, with significant increases observed in each cycle. For individuals aged 45 and older, the overall time trend of BRI in the HRS data was statistically significant (*P* = 0.003). However, in the HRS data, although the average BRI increased from 5.028 (95% CI, 4.986–5.071) to 5.113 (95% CI, 5.069–5.158), using the average BRI from the 2004–2005 cycle as a reference, the change range was 0.147–0.085, and significant increases were observed in each cycle, but the overall time trend did not reach statistical significance.

Additionally, the time trend of BRI in the HRS and ELSA datasets was stratified by sociodemographic factors (Supplementary Figures 1, 2 and Supplementary Tables 2, 3). Both datasets show that BRI increases with age and cycle duration. Overall, women have higher BRI than men, with an increasing trend over time. By education level, adults with a high school diploma or higher have the lowest BRI, while those with less than a high school education consistently exhibit the highest BRI. Moreover, individuals who drink alcohol and smoke have relatively lower BRI, while those with hypertension, diabetes, and cardiovascular disease have relatively lower average BRI. Furthermore, compared to individuals who never exercise, those who engage in physical activity have relatively lower average BRI.

### 3.3 Association between estimated BRI and COPD

To address potential multicollinearity issues, we selected 14 significant variables from the univariate analysis (Table 1) for variance inflation factor (VIF) analysis, including BRI, age, education level (education), marital status (marital), alcohol consumption (drink), smoking status (smoke), hypertension, cardiovascular disease (CVD), vigorous physical activity, moderate physical activity, mild physical activity, smoking quantity per day, body mass index (BMI), and CESD score (Supplementary Table 4). The VIF results indicated that all variables had VIF values below 5, suggesting no multicollinearity issues.

Then three multivariable regression models (Table 2) were constructed to further examine the relationship between BRI and COPD. Model 1 is the unadjusted baseline model, Model 2 adjusts for age, education, alcohol consumption, smoking, hypertension, and cardiovascular disease, while Model 3 further adjusts for all variable. The results showed that in the unadjusted ELSA Model 1, each one-unit increase in BRI was associated with a 9.7% increase in the odds of COPD (OR = 1.097, *P* = 0.004). However, in Model 2, after adjusting for age, education, alcohol consumption, smoking, hypertension, and cardiovascular disease, this relationship was no longer significant. In contrast, in Model 3, after fully adjusting for all variable, each one-unit increase in BRI was associated with a 19.3% increase in the odds of COPD (OR = 1.193, *P* = 0.001).

**TABLE 2:** Multivariable logistic regression models for the association between BRI and COPD.

Variables		Crude Model1^a^		Model2^b^		Model3^c^	
		OR (95%CI)		P-value		OR (95%CI)	
ELSA	BRI	1.097(1.030–1.167)	0.004	1.057(0.988–1.129)	0.102	1.193(1.074–1.321)	0.001
	Categories						
	Q1(1.332–3.879)	Reference		Reference		Reference	
	Q2(3.879–4.839)	0.934(0.674–1.294)	0.681	0.880(0.631–1.224)	0.447	1.197(0.821–1.744)	0.35
	Q3(4.839–5.977)	1.095(0.800–1.501)	0.572	0.992(0.719–1.370)	0.962	1.615(1.043–2.508)	0.032
	Q4(5.977–14.798)	1.341(0.993–1.818)	0.057	1.109(0.811–1.521)	0.518	2.008(11.188–3.406)	0.009
	P^d^ for trend	0.0306		0.371		0.006	
HRS	BRI	1.100(1.059–1.142)	<0.001	1.097(1.053–1.142)	<0.001	1.160(1.094–1.228)	<0.001
	Categories						
	Q1(1.222-4.194)	Reference		Reference		Reference	
	Q2(4.194–5.287)	0.997(0.776–1.282)	0.984	0.986(0.762–1.276)	0.914	1.275(0.948–1.716)	0.108
	Q3(5.287–6.757)	0.971(0.754–1.249)	0.816	0.941(0.725–1.222)	0.65	1.401(0.985–1.997)	0.061
	Q4(6.757–18.994)	1.443(1.144–1.825)	0.002	1.365(1.065–1.755)	0.014	1.942(1.297–2.915)	0.001
	P for trend	0.003		0.019		0.002	

a, Without adjustment,

b, Adjusted for age, education, alcohol, smoke, hypertension and CVD

c, Adjusted for all variables,

d, *P* for trend is calculated by converting the quartiles of BRI into level variables, assigning values of 0, 1, 2, and 3, and then inputting the level variables into the regression model.

In the HRS data, in the unadjusted Model 1, each one-unit increase in BRI was associated with a 10% increase in the odds of COPD (OR = 1.10, *P* < 0.001). In the partially adjusted Model 2, each one-unit increase in BRI was associated with a 9.7% increase in COPD risk (OR = 1.097, *P* < 0.001). In the fully adjusted Model 3, each one-unit increase in BRI was associated with a 16.0% increase in the odds of COPD (OR = 1.160, *P* < 0.001).

### 3.4 The relationship between body roundness index and newly diagnosed COPD

Univariate Cox regression analysis of the HRS cohort (6,048 participants) and the ELSA cohort (4,984 participants) for baseline participants without COPD showed that age, smoking quantity per day, and Body Roundness Index (BRI) were significant risk factors for incident COPD (*P*<0.05) (Supplementary Table 5). Additionally, a higher education level was associated with a lower hazard ratio (HR) for COPD in both populations (*P*<0.05). Smokers, individuals with emotional/neurological/mental issues, cardiovascular disease (CVD), and higher CESD scores exhibited a significantly higher HR for COPD (*P*<0.05). Moreover, varying levels of physical activity were found to significantly reduce the HR for COPD (*P*<0.05).Interestingly, compared to the underweight group, the HRS data indicated that overweight and obese individuals had a significantly lower HR for COPD (overweight group: HR, 0.31, 95% CI, 0.15–0.63, *P* = 0.001; obese group: HR, 0.35, 95% CI,0.18–0.72, P = 0.004). In the ELSA cohort, the overweight group exhibited similar findings to HRS (HR, 0.31, 95% CI,0.11–0.84, *P* = 0.021).

After excluding collinearity (Supplementary Table 6), three Cox regression models were constructed to assess the relationship between BRI and newly diagnosed COPD. Model 1 was the unadjusted baseline model, Model 2 adjusted for BRI, education, smoking status, cardiovascular disease (CVD), and daily smoking quantity, while Model 3 further adjusted for all covariates.

The results showed (Table 3) that in the unadjusted ELSA Model 1, each one-unit increase in BRI was associated with a 14.9% increase in the risk of COPD (HR = 1.149, 95% CI: 1.078–1.224, *P* < 0.001). In Model 2, after adjusting for BRI, education, smoking status, CVD, and daily smoking quantity, each one-unit increase in BRI was associated with a 12.4% increase in the risk of COPD (HR = 1.124, 95% CI: 1.051–1.202, *P* = 0.001). In the fully adjusted Model 3, each one-unit increase in BRI was associated with a 14.7% increase in the risk of COPD (HR = 1.147, 95% CI: 1.034–1.273, *P* = 0.009).

**TABLE 3:** Multivariable COX regression models for the association between BRI and Newly diagnosed COPD.

Variables		Crude Model1^a^		Model2^b^		Model3^c^	
		HR(95%CI)	P value	HR(95%CI)	P value	HR(95%CI)	P value
ELSA	BRI	1.149(1.078–1.224)	<0.001	1.124(1.051–1.202)	0.001	1.147(1.034–1.273)	0.009
	Categories						
	Q1(1.332–3.863)	Reference		Reference		Reference	
	Q2(3.863–4.813)	1.378(0.952–1.995)	0.089	1.271(0.878–1.840)	0.205	1.677(1.097–2.564)	0.017
	Q3(4.813–5.953)	1.631(1.138–2.338)	0.008	1.530(1.065–2.198)	0.021	2.151(1.315–3.519)	0.002
	Q4(5.953–14.798)	2.018(1.426–2.856)	<0.001	1.747(1.228–2.484)	0.002	2.428(1.378–4.278)	0.002
	P^d^ for trend	<0.001		0.001		0.003	
HRS	BRI	1.061(1.022–1.102)	0.002	1.068(1.027–1.110)	0.001	1.114(1.054–1.177)	<0.001
	Categories						
	Q1(1.222–4.194)	Reference		Reference		Reference	
	Q2(4.194–5.273)	1.007(0.792–1.281)	0.954	0.985(0.774–1.254)	0.902	1.308(0.989–1.732)	0.06
	Q3(5.273–6.700)	1.102(0.869–1.397)	0.424	1.057(0.832–1.343)	0.648	1.650(1.184–2.298)	0.003
	Q4(6.700–18.994)	1.397(1.115—1.751)	0.004	1.412(1.121–1.779)	0.003	2.286(1.563–3.344)	<0.001
	P for trend	0.002		0.003		<0.001	

a, Without adjustment,

b, Adjusted for age, education, alcohol, smoke, hypertension and CVD

c, Adjusted for all variables,

d, P for trend is calculated by converting the quartiles of BRI into level variables, assigning values of 0, 1, 2, and 3, and then inputting the level variables into the regression model.

In the HRS data, in the unadjusted Model 1, each one-unit increase in BRI was associated with a 6.1% increase in the risk of COPD (HR = 1.061, 95% CI: 1.022–1.102, *P* = 0.002). In the partially adjusted Model 2, each one-unit increase in BRI was associated with a 6.8% increase in the risk of COPD (HR = 1.068, 95% CI: 1.027–1.110, *P* = 0.001). In the fully adjusted Model 3, each one-unit increase in BRI was associated with an 11.4% increase in the risk of COPD (HR = 1.114, 95% CI: 1.054–1.177, *P* < 0.001).

### 3.5 Analysis of the nonlinear relationship between BRI and COPD

**FIGURE 2:**
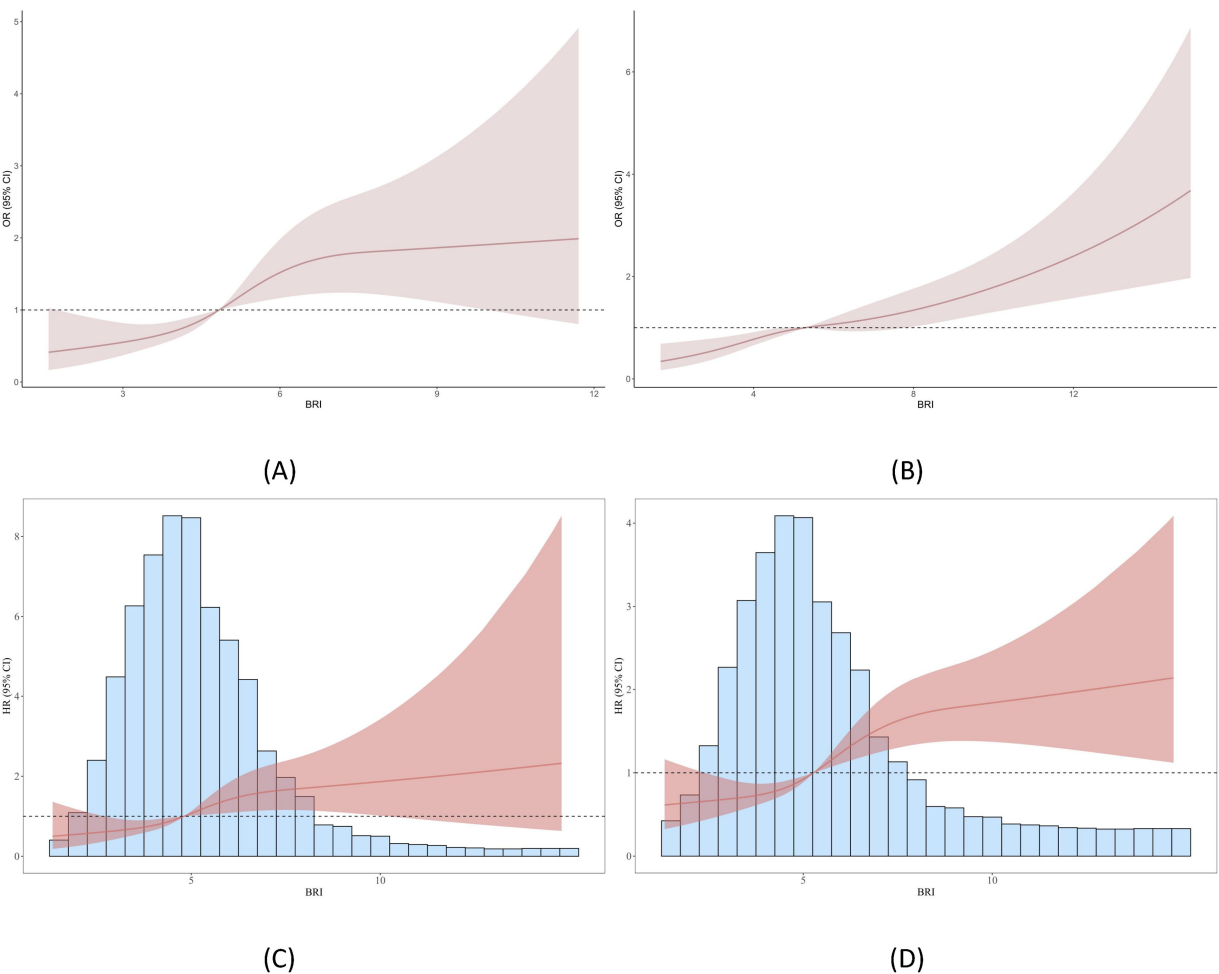
Smooth curve fitting (RCS analysis) between BRI and COPD. **(A)** Shows the relationship between BRI and COPD in the ELSA dataset; **(B)** illustrates the relationship between BRI and COPD in the HRS dataset; **(C)** presents the relationship between BRI and newly diagnosed COPD in the ELSA dataset; and **(D)** demonstrates the relationship between BRI and newly diagnosed COPD in the HRS dataset.

Based on the Model 3 of BRI and COPD and the Model 3 of BRI and newly diagnosed COPD, we constructed a restricted cubic spline (RCS) analysis, as shown in Figure 2. The results of the analysis indicated that in the ELSA data, with the increase in BRI, the risk of COPD significantly increased (*P* for overall = 0.001, *P* for nonlinear = 0.050). The threshold effect analysis revealed a threshold of 4.838 in the ELSA data; this trend was also observed in the HRS data (*P* for overall < 0.001, *P* for nonlinear = 0.272), with a threshold of 5.287. In the analysis of newly diagnosed COPD, the ELSA data also showed that with the increase in BRI, the risk of developing COPD increased (*P* for overall = 0.016, *P* for nonlinear = 0.120), with a threshold effect analysis result of 4.813. This trend was similarly observed in the HRS data (*P* for overall < 0.001, *P* for nonlinear = 0.027), with a threshold effect analysis result of 5.273.

**FIGURE 3:**
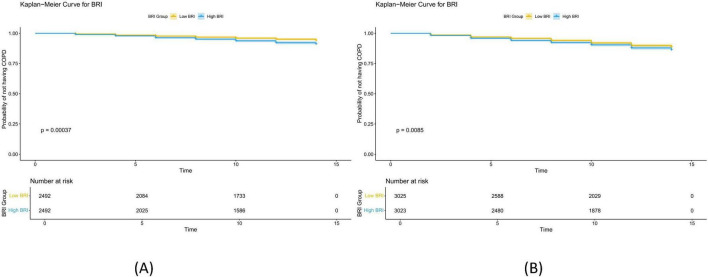
Kaplan Meier curves of BRI and newly diagnosed COPD. **(A)** Shows the Kaplan Meier curves of BRI and newly diagnosed COPD in ELSA data. The samples were divided into low BRI group and high BRI group according to the threshold of 4.813; **(B)** shows the Kaplan Meier curves of BRI and newly diagnosed COPD in HRS data. The samples were divided into low BRI group and high BRI group according to the threshold of 5.273. The results indicate that with the increase of BRI, the risk of developing new COPD significantly increases.

The Kaplan-Meier (KM) curve (Figure 3) demonstrates that in both datasets, there is a significant difference in COPD prevalence between the low BRI and high BRI groups.

### 3.6 Sensitivity analysis

We performed sensitivity analyses to assess the stability and external validity of the association results. After removing the 5% extreme values of BRI at both ends, the analysis results (Supplementary Table 7) showed that a significant positive association between BRI and COPD remained [ELSA: OR (95% CI) = 1.348 (1.156–1.574), *P* < 0.001; HRS: OR (95% CI) = 1.172 (1.069–1.285), *P* = 0.001). Furthermore, as BRI increased, the risk of COPD significantly increased in both datasets (ELSA, *P* for trend = 0.003; HRS, *P* for trend = 0.007). Supplementary Table 8 shows the relationship between BRI and incident COPD after removing the 5% extreme values of BRI at both ends. The results indicated that the relationship between BRI and incident COPD remained consistent in both the ELSA (HR (95% CI) = 1.220 (1.036–1.437), *P* = 0.017) and HRS [HR (95% CI) = 1.199 (1.099–1.308), *P* < 0.001] datasets. The results after multiple imputation for missing covariates (Supplementary Table 9) showed that in the ELSA, OR (95% CI) = 1.139 (1.039–1.247), *P* = 0.005; in the HRS, OR (95% CI) = 1.144 (1.088–1.201), *P* < 0.001. Supplementary Table 10 displays the relationship between BRI and incident COPD, showing HR (95% CI) = 1.108 (1.006–1.221), *P* = 0.037 in the ELSA and HR (95% CI) = 1.081 (1.030–1.135), *P* = 0.002 in the HRS. The results of the cumulative smoking exposure model for COPD are presented in Supplementary Table 11. As expected, the incidence of COPD was significantly higher in individuals with cumulative smoking exposure compared to those without exposure (*P* < 0.05). In addition, Supplementary Figures 6, 7 and Supplementary Table 12 demonstrate that BRI consistently showed better predictive performance for COPD than LAP and VAI.

### 3.7 Subgroup analysis

The subgroup analysis results show (Supplementary Figure 3) that in the ELSA dataset, BRI is significantly positively associated with COPD in most subgroups, and there is a significant difference in the response to COPD risk across different age groups at varying BRI levels (*P* for interaction = 0.027). Unlike ELSA, in the HRS dataset, different levels of moderate activity show a significant interaction with BRI in relation to COPD risk (*P* for interaction = 0.042). Subgroup analyses shown in Supplementary Figures 4, 5 indicate that there are significant differences in the response to COPD risk across different BMI groups at varying BRI levels (ELSA, *P* for interaction = 0.014; HRS, *P* for interaction = 0.036).

## 4 Discussion

This cohort study aimed to describe the time trends of the Body Roundness Index (BRI) in populations aged at least 45 years from the HRS data (2006–2019) and ELSA data (2004–2013), and to explore the relationship between BRI and COPD risk as well as newly diagnosed COPD. Notably, during the 14-year research period of the HRS, BRI showed a stable upward trend, especially in the population aged 45–60 years, where this trend was more pronounced. The results indicated that a higher BRI may increase the risk of COPD [ELSA: OR (95%CI) = 1.193 (1.074–1.321), *p* = 0.001; HRS: OR (95%CI) = 1.160 (1.094–1.228), P < 0.001]. Moreover, a higher BRI was also closely associated with an increased risk of newly diagnosed COPD [ELSA: HR (95%CI) = 1.147 (1.034–1.273), *P* = 0.009; HRS: HR (95%CI) = 1.114 (1.054–1.177), *P* < 0.001]. Further RCS analysis revealed a “J-shaped” relationship between BRI and COPD, indicating that with the increase of BRI, the risk of newly diagnosed COPD rises significantly. Through threshold effect analysis, we identified the optimal cutoff value for BRI. Additionally, sensitivity analysis verified the stability and consistency of the results.

Existing studies have shown that obesity significantly impacts lung function, primarily through the effect of fat tissue on respiratory mechanics (35–38). Abdominal obesity reduces lung compliance and decreases lung capacity, while visceral fat impairs lung function by altering diaphragm structure and limiting its movement (39, 40). Visceral fat is more harmful than subcutaneous fat due to its contribution to multiple diseases, including COPD (41, 42). Studies have demonstrated that visceral fat, measured by computed tomography (CT), is a significant independent risk factor for COPD, while traditional measures like BMI and waist circumference may not fully capture its effect (43, 44). BRI, as a comprehensive assessment of abdominal fat distribution, particularly visceral fat, offers a more precise reflection of fat accumulation than traditional indices like BMI and LAP (45–47). Our study shows that as BRI increases, COPD risk and prevalence also increase, likely due to changes associated with obesity. Excessive visceral fat exacerbates airway inflammation through the secretion of pro-inflammatory factors (e.g., TNF-α, IL-6, CRP), which are crucial in COPD pathogenesis (17, 18, 48, 49). Chronic inflammation and oxidative stress, driven by visceral fat, impair lung cell repair and accelerate airway remodeling, leading to progressive lung dysfunction (50). Furthermore, visceral fat may elevate the diaphragm, reducing thoracic volume, restricting lung expansion, and increasing respiratory muscle burden, which worsens COPD symptoms (51). Additionally, visceral fat accumulation may influence hormone levels (e.g., cortisol and estrogen), with elevated cortisol suppressing immune function and increasing infection risk, while estrogen changes may affect lung inflammation and repair mechanisms (52–54). Increased abdominal fat may also disrupt the autonomic nervous system, enhancing airway hyperreactivity, causing bronchospasm and dyspnea, and aggravating COPD symptoms (55–57).

In our analysis, we observed a “J-shaped” relationship between Body Roundness Index (BRI) and Chronic Obstructive Pulmonary Disease (COPD), where lower BRI values were associated with a reduced risk of COPD. This finding is particularly noteworthy, as lower BRI typically reflects lower body weight and reduced abdominal fat, which may represent a distinct COPD phenotype, such as cachectic emphysema (58). In this phenotype, individuals often experience severe muscle wasting, malnutrition, and significant weight loss, which may independently contribute to lung function decline without significant abdominal fat accumulation (59). Unlike individuals with higher BRI values, who primarily face risks related to visceral fat accumulation, those with lower BRI may develop COPD through mechanisms such as muscle wasting and metabolic disturbances (60). These individuals may experience reduced respiratory muscle strength, further exacerbating lung function impairment, and they may exhibit different disease progression, potentially leading to poorer clinical outcomes despite lower abdominal fat.

The mechanisms driving COPD in individuals with low versus high BRI are likely distinct (61). High BRI is closely associated with increased visceral fat accumulation, which plays a critical role in the pathogenesis of COPD by promoting systemic inflammation, oxidative stress, and disrupting respiratory mechanics (62). These mechanisms are typically linked to excessive abdominal fat, where the accumulation of visceral fat worsens airway inflammation and impairs lung function (17). In contrast, low BRI individuals, characterized by lower abdominal fat and possibly reduced muscle mass, may develop COPD through a combination of systemic inflammation, muscle wasting, and nutritional deficiencies. The lack of sufficient muscle mass, compounded by poor nutrition, leads to decreased respiratory muscle strength, making it more difficult to maintain lung function, even in the absence of significant body fat (23, 63). These individuals may exhibit a distinct COPD phenotype, characterized by more rapid functional decline and worse clinical outcomes, even without significant abdominal fat accumulation.

Additionally, an important observation in the ELSA data was the change in the significance of the association between BRI and COPD when comparing the semi-adjusted model (Model 2) with the fully adjusted model (Model 3).In Model 2, BRI’s association with COPD was not significant (*p* = 0.102), but in Model 3, where more comprehensive covariates–including mental health, physical activity, and BMI–were considered, the association regained significance (*p* = 0.001). This suggests that some sociodemographic factors may attenuate the effect of BRI in the partially adjusted model, while the inclusion of additional health-related variables in the fully adjusted model better accounts for confounding and reveals the independent effect of BRI. This pattern highlights the complexity of factors influencing COPD risk and raises the possibility of mediating or interacting effects between BRI and other lifestyle or health-related factors, such as smoking, physical activity, and psychological status, which warrants further investigation in future studies.

Subgroup analysis results show that, whether it is vigorous, moderate, or mild exercise, people who engage in exercise at least once a week have significantly higher COPD risks, and this trend is consistently observed in both HRS and ELSA data. Furthermore, as the exercise intensity increases, the risk of COPD rises significantly. This finding contradicts our usual expectation that exercise benefits health, suggesting that the relationship between exercise intensity and COPD risk may be significantly influenced by individual health status (64, 65). For individuals in good health, moderate exercise helps maintain good lung function and cardiovascular health, thereby reducing the occurrence of COPD (65). However, for individuals with an existing COPD risk, especially those engaging in vigorous and high-frequency exercise, exercise may lead to physical exhaustion, increase the burden on the respiratory system, and worsen symptoms or induce the onset of COPD. In high-risk populations (e.g., the elderly or those with pre-existing respiratory issues), excessive exercise intensity may backfire, causing adverse effects (66, 67).

Moreover, exercise frequency is also an important factor influencing COPD risk. In both datasets, although individuals who exercise at least once a week exhibit a significant increase in COPD risk with increasing exercise intensity, low-frequency exercise may also result in the lack of health benefits (67). The possible explanation is that the combination of frequency and intensity is crucial for COPD prevention. Vigorous exercise performed at low frequency may fail to significantly improve lung function and may even have negative effects (68, 69). Therefore, exercise interventions should be more personalized, adjusting exercise frequency and intensity based on the health status and physical abilities of different populations.

Interestingly, the analysis results also show that individuals with higher education levels have significantly higher COPD risks and incidence in both datasets, compared to other groups. Furthermore, overweight individuals also have a significantly higher risk of new COPD compared to those of normal weight (70). These results suggest that, in addition to exercise intensity and frequency, factors such as socioeconomic status, mental health, and body weight may also play significant roles in the development of COPD (71).

However, this study relies on cross-sectional and longitudinal data, which limits the ability to draw causal inferences. While we have explored the relationship between Body Roundness Index (BRI) and Chronic Obstructive Pulmonary Disease (COPD) through multiple waves of follow-up, the influence of potential confounders cannot be fully excluded. Additionally, this study used data from two population-based cohorts (ELSA and HRS) in the UK and the US, which predominantly include older adults and individuals from higher-income backgrounds. Therefore, the findings may be more applicable to these specific groups, and due to the limitations in age and income levels of the participants in these datasets, the generalizability of the results may be constrained, particularly for younger, lower-income, or more ethnically diverse populations. In these populations, obesity types, visceral fat distribution, and the mechanisms underlying COPD may differ from the sample in our study. Therefore, future research should include more diverse populations to further validate the predictive ability and applicability of BRI in these groups.

Another important limitation of this study is that the diagnosis of COPD was based on self-reported physician diagnosis. This approach may introduce potential information bias, as spirometry-based measures such as FEV1/FVC ratios or detailed respiratory symptom assessments were not available in either the HRS or ELSA datasets. As a result, we were unable to conduct sensitivity analyses using objective lung function data or symptom-based definitions of COPD. Nevertheless, we performed a series of additional sensitivity analyses, including cumulative smoking exposure and subgroup analyses, and the results consistently supported the robustness of our main findings. Future studies incorporating spirometry-confirmed COPD diagnoses and detailed clinical symptom assessments are warranted to further validate these associations.

## 5 Conclusion

This study shows that BRI, as a new tool for COPD risk assessment, can accurately reflect the occurrence and progression of COPD, especially in individuals with more visceral fat accumulation, which has important clinical significance. The type, intensity and frequency of exercise play an important role in the prevention and management of COPD. In the future, individualized exercise intervention programs should be formulated according to individual health status to effectively reduce the incidence of COPD and improve the health status of patients.

## Data Availability

The raw data supporting the conclusions of this article are publicly available. Data from the Health and Retirement Study (HRS) can be accessed at https://hrs.isr.umich.edu/, and data from the English Longitudinal Study of Ageing (ELSA) can be accessed at https://www.elsa-project.ac.uk/. Access to these datasets requires registration and approval from the respective data providers.
