# Supplementary material for: Body roundness index and its role in predicting COPD risk: insights from the English Longitudinal Study of Aging and the health and retirement study
Source: Front Med (Lausanne). 2025 Oct 16;12:1670309. doi: 10.3389/fmed.2025.1670309 (PMC12571653; doi:10.3389/fmed.2025.1670309)
Supplement: Supplementary file 1 [file Supplementary_file_1.docx]

| **Supplementary Table 1 Magnitude of Changes in Mean BRI for Subsequent the Health and Retirement Study and the English Longitudinal Study on Ageing Cycles^a^ .** | | | | |
| --- | --- | --- | --- | --- |
| **Survey year** | **Mean (SE) [95% CI]** | **Difference** | **P value for difference in BRI^b^** | **Overall P value for trend^c^** |
| the Health and Retirement Study | | | | |
| 2006-2007 | 5.642(0.025)[5.594-5.691] | 0 [Reference] | NA |  |
| 2008-2009 | 5.804(0.028)[5.750-5.859] | 0.162 | <0.001 | 0.003 |
| 2010-2011 | 5.871(0.025)[5.821-5.921] | 0.229 | <0.001 |  |
| 2012-2013 | 5.884(0.026)[5.834-5.934] | 0.242 | <0.001 |  |
| 2014-2015 | 6.065(0.054)[5.959-6.171] | 0.423 | <0.001 |  |
| 2016-2017 | 6.217(0.057)[6.104-6.330] | 0.575 | <0.001 |  |
| 2018-2019 | 6.224(0.062)[6.102-6.346] | 0.582 | <0.001 |  |
| the English Longitudinal Study on Ageing | | | | |
| 2004-2005 | 5.028(0.022)[4.986-5.071] | 0 [Reference] | NA |  |
| 2008-2009 | 5.175(0.022)[5.132-5.218] | 0.147 | <0.001 | 1.00 |
| 2012-2013 | 5.113(0.023)[5.069-5.158] | 0.085 | 0.007 |  |
| *Abbreviations: BRI, body roundness index; NA, not applicable.*  *a, The ELSA and HRS populations aged 45 and above who met the inclusion criteria covered data from each cycle.*  *b, P value for difference in mean BRI was calculated using the linear combinations of parameters.*  *c, P value for overall trend was calculated using the Mann-Kendall trend test.* | | | | |

| **Supplementary Table 4 Variance Inflation Factor** | | |
| --- | --- | --- |
| **Characteristic** | **VIF_ELSA_** | **VIF_HRS_** |
| **BRI** | 2.906 | 2.457 |
| **Age** | 1.388 | 1.428 |
| **Education** | 1.157 | 1.150 |
| **Marital** | 1.182 | 1.162 |
| **Alcohol** | 1.074 | 1.100 |
| **Smoke** | 2.039 | 2.619 |
| **Hypertension** | 1.103 | 1.116 |
| **CVD** | 1.119 | 1.137 |
| **Vigorous activity** | 1.221 | 1.331 |
| **Moderate activity** | 1.633 | 1.822 |
| **Mild activity** | 1.377 | 1.542 |
| **Smoking quantity(per day)** | 1.955 | 2.446 |
| **BMI** | 2.989 | 2.485 |
| **CESD** | 1.224 | 1.257 |
| *CVD, cardiovascular disease ,BRI, body roundness index; CESD, the Center for Epidemiologic Studies Depression Scale (CES‐D).* | | |

| **Supplementary Table 5 Single factor Cox regression** | | | | | | | | |
| --- | --- | --- | --- | --- | --- | --- | --- | --- |
| **Characteristic** | **ELSA** | | | | **HRS** | | | |
|  | **β** | **S.E** | **P** | **HR (95%CI)** | **β** | **S.E** | **P** | **HR (95%CI)** |
| **Age** | 0.02 | 0.01 | 0.012 | 1.02 (1.01 ~ 1.03) | 0.02 | 0 | <0.001 | 1.02 (1.01 ~ 1.03) |
| **Smoking quantity(per day)** | 0.05 | 0.01 | <0.001 | 1.06 (1.04 ~ 1.07) | 0.04 | 0 | <0.001 | 1.04 (1.04 ~ 1.05) |
| **BRI** | 0.14 | 0.03 | <0.001 | 1.15 (1.08 ~ 1.22) | 0.06 | 0.02 | 0.002 | 1.06 (1.02 ~ 1.10) |
| **Sex** |  |  |  |  |  |  |  |  |
| Female |  |  |  | (Reference) |  |  |  | (Reference) |
| Male | 0.29 | 0.12 | 0.014 | 1.34 (1.06 ~ 1.70) | -0.08 | 0.09 | 0.348 | 0.92 (0.78 ~ 1.09) |
| **Race** |  |  |  |  |  |  |  |  |
| White |  |  |  | (Reference) |  |  |  | (Reference) |
| Other | 0.54 | 0.71 | 0.45 | 1.71 (0.43 ~ 6.87) | 0 | 0.11 | 0.994 | 1.00 (0.81 ~ 1.24) |
| **Education** |  |  |  |  |  |  |  |  |
| less than high school |  |  |  | (Reference) |  |  |  | (Reference) |
| high school | -0.36 | 0.13 | 0.004 | 0.69 (0.54 ~ 0.89) | -0.32 | 0.1 | 0.002 | 0.72 (0.59 ~ 0.89) |
| Higher | -0.86 | 0.21 | <0.001 | 0.42 (0.28 ~ 0.64) | -0.77 | 0.13 | <0.001 | 0.47 (0.36 ~ 0.61) |
| **Marital** |  |  |  |  |  |  |  |  |
| Never married |  |  |  | (Reference) |  |  |  | (Reference) |
| Married | 0.62 | 0.42 | 0.136 | 1.86 (0.82 ~ 4.19) | -0.41 | 0.21 | 0.054 | 0.67 (0.44 ~ 1.01) |
| Other | 1.11 | 0.42 | 0.008 | 3.05 (1.34 ~ 6.96) | 0.07 | 0.22 | 0.742 | 1.07 (0.70 ~ 1.64) |
| **Alcohol** |  |  |  |  |  |  |  |  |
| No |  |  |  | (Reference) |  |  |  | (Reference) |
| Yes | -0.36 | 0.19 | 0.058 | 0.70 (0.48 ~ 1.01) | -0.26 | 0.08 | 0.002 | 0.77 (0.66 ~ 0.91) |
| **Smoke** |  |  |  |  |  |  |  |  |
| No |  |  |  | (Reference) |  |  |  | (Reference) |
| Yes | 1.46 | 0.12 | <0.001 | 4.31 (3.38 ~ 5.50) | 1.27 | 0.09 | <0.001 | 3.58 (3.00 ~ 4.27) |
| **Hypertension** |  |  |  |  |  |  |  |  |
| No |  |  |  | (Reference) |  |  |  | (Reference) |
| Yes | 0.08 | 0.12 | 0.506 | 1.09 (0.85 ~ 1.38) | 0.18 | 0.08 | 0.028 | 1.20 (1.02 ~ 1.42) |
| **Diabetes mellitus** |  |  |  |  |  |  |  |  |
| No |  |  |  | (Reference) |  |  |  | (Reference) |
| Yes | 0 | 0.24 | 0.991 | 1.00 (0.62 ~ 1.62) | 0.07 | 0.11 | 0.555 | 1.07 (0.86 ~ 1.33) |
| **Cancer** |  |  |  |  |  |  |  |  |
| No |  |  |  | (Reference) |  |  |  | (Reference) |
| Yes | 0.1 | 0.24 | 0.694 | 1.10 (0.68 ~ 1.77) | 0.19 | 0.12 | 0.125 | 1.21 (0.95 ~ 1.54) |
| **Emotional/neurological/mental issues** |  |  |  |  |  |  |  |  |
| No |  |  |  | (Reference) |  |  |  | (Reference) |
| Yes | 0.43 | 0.19 | 0.022 | 1.53 (1.06 ~ 2.20) | 0.51 | 0.11 | <0.001 | 1.67 (1.35 ~ 2.06) |
| **CVD** |  |  |  |  |  |  |  |  |
| No |  |  |  | (Reference) |  |  |  | (Reference) |
| Yes | 0.49 | 0.14 | <0.001 | 1.64 (1.25 ~ 2.13) | 0.52 | 0.09 | <0.001 | 1.69 (1.41 ~ 2.02) |
| **Vigorous ctivity** |  |  |  |  |  |  |  |  |
| Never |  |  |  | (Reference) |  |  |  | (Reference) |
| 1–3 times per month | -0.36 | 0.2 | 0.065 | 0.70 (0.47 ~ 1.02) | -0.14 | 0.16 | 0.367 | 0.87 (0.64 ~ 1.18) |
| Once a week | -0.44 | 0.21 | 0.034 | 0.65 (0.43 ~ 0.97) | -0.33 | 0.16 | 0.036 | 0.72 (0.52 ~ 0.98) |
| At least once a week | -0.8 | 0.18 | <0.001 | 0.45 (0.32 ~ 0.64) | -0.49 | 0.11 | <0.001 | 0.61 (0.49 ~ 0.77) |
| Every day |  |  |  |  | -0.22 | 0.26 | 0.382 | 0.80 (0.49 ~ 1.32) |
| **Moderate activity** |  |  |  |  |  |  |  |  |
| Never |  |  |  | (Reference) |  |  |  | (Reference) |
| 1–3 times per month | -0.45 | 0.22 | 0.04 | 0.64 (0.41 ~ 0.98) | -0.36 | 0.17 | 0.032 | 0.70 (0.50 ~ 0.97) |
| Once a week | -0.61 | 0.19 | 0.001 | 0.55 (0.38 ~ 0.78) | -0.29 | 0.14 | 0.03 | 0.74 (0.57 ~ 0.97) |
| At least once a week | -1.22 | 0.15 | <0.001 | 0.29 (0.22 ~ 0.40) | -0.54 | 0.11 | <0.001 | 0.58 (0.47 ~ 0.72) |
| Every day |  |  |  |  | -0.53 | 0.16 | 0.001 | 0.59 (0.43 ~ 0.81) |
| **Mild activity** |  |  |  |  |  |  |  |  |
| Never |  |  |  | (Reference) |  |  |  | (Reference) |
| 1–3 times per month | -0.64 | 0.36 | 0.074 | 0.53 (0.26 ~ 1.06) | -0.24 | 0.21 | 0.259 | 0.78 (0.52 ~ 1.20) |
| Once a week | -0.13 | 0.24 | 0.601 | 0.88 (0.55 ~ 1.41) | -0.43 | 0.17 | 0.013 | 0.65 (0.46 ~ 0.91) |
| At least once a week | -0.91 | 0.21 | <0.001 | 0.40 (0.27 ~ 0.60) | -0.58 | 0.16 | <0.001 | 0.56 (0.41 ~ 0.77) |
| Every day |  |  |  |  | -0.48 | 0.19 | 0.011 | 0.62 (0.43 ~ 0.90) |
| **CESD** |  |  |  |  |  |  |  |  |
| 0 |  |  |  | (Reference) |  |  |  | (Reference) |
| 1 | 0.16 | 0.16 | 0.334 | 1.17 (0.85 ~ 1.60) | 0.2 | 0.11 | 0.073 | 1.22 (0.98 ~ 1.52) |
| 2 | 0.34 | 0.21 | 0.098 | 1.41 (0.94 ~ 2.12) | 0.54 | 0.13 | <0.001 | 1.71 (1.33 ~ 2.20) |
| 3 | 0.86 | 0.21 | <0.001 | 2.37 (1.58 ~ 3.54) | 0.48 | 0.16 | 0.003 | 1.62 (1.17 ~ 2.23) |
| 4 | 0.9 | 0.25 | <0.001 | 2.45 (1.50 ~ 4.02) | 0.62 | 0.19 | 0.001 | 1.87 (1.28 ~ 2.73) |
| 5 | 0.9 | 0.3 | 0.002 | 2.45 (1.37 ~ 4.38) | 0.1 | 0.26 | 0.692 | 1.11 (0.67 ~ 1.84) |
| 6 | 0.69 | 0.32 | 0.032 | 1.99 (1.06 ~ 3.71) | 0.39 | 0.24 | 0.115 | 1.47 (0.91 ~ 2.37) |
| 7 | 0.82 | 0.37 | 0.026 | 2.28 (1.11 ~ 4.69) | 0.55 | 0.28 | 0.046 | 1.73 (1.01 ~ 2.97) |
| 8 | 1.4 | 0.37 | <0.001 | 4.04 (1.96 ~ 8.32) | 1.27 | 0.25 | <0.001 | 3.58 (2.19 ~ 5.85) |
| **BMI** |  |  |  |  |  |  |  |  |
| Underweight |  |  |  | (Reference) |  |  |  | (Reference) |
| Normal | -1.14 | 0.51 | 0.027 | 0.32 (0.12 ~ 0.88) | -0.86 | 0.36 | 0.018 | 0.42 (0.21 ~ 0.86) |
| Overweight | -1.18 | 0.51 | 0.021 | 0.31 (0.11 ~ 0.84) | -1.17 | 0.36 | 0.001 | 0.31 (0.15 ~ 0.63) |
| Obesity | -0.94 | 0.51 | 0.067 | 0.39 (0.14 ~ 1.07) | -1.04 | 0.36 | 0.004 | 0.35 (0.18 ~ 0.72) |
| *CVD, cardiovascular disease, BRI, body roundness index; CESD, the Center for Epidemiologic Studies Depression Scale (CES‐D).* | | | | | | | | |

| **Supplementary Table 6 Variance Inflation Factor (cox regression)** | | |
| --- | --- | --- |
| **Characteristic** | **VIF_ELSA_** | **VIF_HRS_** |
| **BRI** | 1.257 | 1.276 |
| **Education** | 1.189 | 1.149 |
| **Smoke** | 1.943 | 2.187 |
| **Smoking quantity(per day)** | 1.850 | 2.091 |
| **Emotional/neurological/mental issues** | 1.088 | 1.197 |
| **Vigorous activity** | 1.090 | 1.079 |
| **Moderate activity** | 1.278 | 1.362 |
| **Mild activity** | 1.613 | 1.904 |
| **CESD** | 1.261 | 1.570 |
| **BMI** | 1.165 | 1.287 |
| **BRI** | 2.816 | 2.496 |
| *CVD, cardiovascular disease, BRI, body roundness index, CESD, the Center for Epidemiologic Studies Depression Scale (CES‐D).* | | |

| **Supplementary Table 7** Multivariate logistic regression models for the association between BRI and COPD, removing 5% outliers in BRI data before and after treatment | | | | |
| --- | --- | --- | --- | --- |
|  | ELSA | | HRS | |
|  | **OR (95%CI)** | **P value** | **OR (95%CI)** | **P value** |
| **BRI** | 1.348(1.156-1.574) | <0.001 | 1.172(1.069-1.285) | 0.001 |
| **Categories** |  |  |  |  |
| **Q1** | **Reference** |  | **Reference** |  |
| **Q2** | 1.274(0.864-1.879) | 0.221 | 1.194(0.884-1.613) | 0.248 |
| **Q3** | 1.602(1.030-2.501) | 0.037 | 1.276(0.899-1.815) | 0.173 |
| **Q4** | 2.147(1.286-3.594) | 0.004 | 1.756(1.178-2.622) | 0.006 |
| **P for trend^a^** | 0.003 |  | 0.007 |  |
| *a, P for trend is calculated by converting the quartiles of BRI into level variables, assigning values of 0, 1, 2, and 3, and then inputting the level variables into the regression model.* | | | | |

| **Supplementary Table 8** Multivariate COX regression models for the association between BRI and Newly diagnosed COPD, removing 5% outliers in BRI data before and after treatment | | | | |
| --- | --- | --- | --- | --- |
|  | ELSA | | HRS | |
|  | **HR (95%CI)** | **P value** | **HR (95%CI)** | **P value** |
| **BRI** | 1.220(1.036-1.437) | 0.017 | 1.199(1.099-1.308) | <0.001 |
| **Categories** |  |  |  |  |
| **Q1** | **Reference** |  | **Reference** |  |
| **Q2** | 1.480(0.963-2.277) | 2.277 | 1.277(0.960-1.699) | 0.093 |
| **Q3** | 1.876(1.158-3.040) | 3.040 | 1.610(1.159-2.237) | 0.005 |
| **Q4** | 2.066(1.193-3.578) | 3.578 | 2.080(1.428-3.031) | <0.001 |
| **P for trend^a^** | 0.009 |  | <0.001 |  |
| *a, P for trend is calculated by converting the quartiles of BRI into level variables, assigning values of 0, 1, 2, and 3, and then inputting the level variables into the regression model.* | | | | |

| **Supplementary Table 9** Multivariate logistic regression models for the association between BRI and COPD, after interpolating missing data | | | | |
| --- | --- | --- | --- | --- |
|  | ELSA | | HRS | |
|  | **OR (95%CI)** | **P value** | **OR (95%CI)** | **P value** |
| **BRI** | 1.139(1.039-1.247) | 0.005 | 1.144(1.088-1.201) | <0.001 |
| **Categories** |  |  |  |  |
| **Q1** | **Reference** |  | **Reference** |  |
| **Q2** | 1.255(0.904-1.740) | 0.174 | 1.341(1.002-1.797) | 0.048 |
| **Q3** | 1.541(1.045-2.277) | 0.029 | 1.294(0.912-1.838) | 0.149 |
| **Q4** | 1.754(1.101-2.797) | 0.018 | 1.954(1.316-2.912) | 0.001 |
| **P for trend^a^** | 0.015 |  | 0.002 |  |
| *a, P for trend is calculated by converting the quartiles of BRI into level variables, assigning values of 0, 1, 2, and 3, and then inputting the level variables into the regression model.* | | | | |

| **Supplementary Table 10** Multivariate COX regression models for the association between BRI and Newly diagnosed COPD, after interpolating missing data | | | | |
| --- | --- | --- | --- | --- |
|  | ELSA | | HRS | |
|  | **HR (95%CI)** | **P value** | **HR (95%CI)** | **P value** |
| **BRI** | 1.108(1.006-1.2210 | 0.037 | 1.081(1.030-1.135) | 0.002 |
| **Categories** |  |  |  |  |
| **Q1** | **Reference** |  | **Reference** |  |
| **Q2** | 1.488(1.021-2.169) | 0.039 | 1.296(0.980-1.713) | 0.069 |
| **Q3** | 1.976(1.279-3.052) | 0.002 | 1.695(1.221-2.353) | 0.002 |
| **Q4** | 2.180(1.316-3.612) | 0.002 | 2.256(1.546-3.293) | <0.001 |
| **P for trend^a^** |  | 0.002 | <0.001 |  |
| *a, P for trend is calculated by converting the quartiles of BRI into level variables, assigning values of 0, 1, 2, and 3, and then inputting the level variables into the regression model.* | | | | |

| **Supplementary Table 11** Calculation and Grouping of Cumulative Smoking Exposure^a^ | | | |
| --- | --- | --- | --- |
| **Data** | **Variable** | **HR(95%CI)** | **P value** |
| **ELSA** | BRI | 1.138(1.020-1.270) | 0.021 |
|  | Cumulative smoking^b^ |  |  |
|  | No | ref |  |
|  | Yes | 3.010(2.245-4.035) | <0.001 |
| **HRS** | BRI | 1.104(1.042-1.169) | 0.001 |
|  | Cumulative smoking |  |  |
|  | No | ref |  |
|  | Yes | 4.200(3.452-5.109) | <0.001 |
| *a,In this model, "smoking status" and "smoking quantity" in Model 3 were replaced with cumulative smoking. b,Cumulative smoking exposure was calculated by averaging the smoking amount from wave 1, wave 2, and wave 3, and then multiplying by the time difference between wave 1 and wave 3 to compute the cumulative exposure."No" indicates the group with no cumulative smoking exposure, while "Yes" indicates the group with cumulative smoking exposure.* | | | |

| **Supplementary Table 12** Time-dependent ROC Analysis of BRI, LAP, and VAI for COPD Prediction | | | | | | |
| --- | --- | --- | --- | --- | --- | --- |
| Variable | TIME | Cases | Survivors | Censored | AUC (%) | SE |
| BRI |  |  |  |  |  |  |
|  | t=4(year) | 28 | 3339 | 642 | 77.86 | 4.11 |
|  | t=8(year) | 100 | 2722 | 1187 | 78.66 | 2.27 |
|  | t=12(year) | 161 | 2041 | 1807 | 79.28 | 1.95 |
| LAP |  |  |  |  |  |  |
|  | t=4(year) | 28 | 3339 | 642 | 76.38 | 4.42 |
|  | t=8(year) | 100 | 2722 | 1187 | 78.26 | 2.35 |
|  | t=12(year) | 161 | 2041 | 1807 | 78.69 | 1.97 |
| VAI |  |  |  |  |  |  |
|  | t=4(year) | 28 | 3339 | 642 | 76.54 | 4.37 |
|  | t=8(year) | 100 | 2722 | 1187 | 78.44 | 2.32 |
|  | t=12(year) | 161 | 2041 | 1807 | 78.6 | 1.98 |
|  |  |  |  |  |  |  |

| **Supplementary Figure 1** Trends in BRI by Socioeconomic Factors Among HRS Participants Aged 45 and Older from 2006 to 2019.  *BRI, body roundness index.* |
| --- |

| 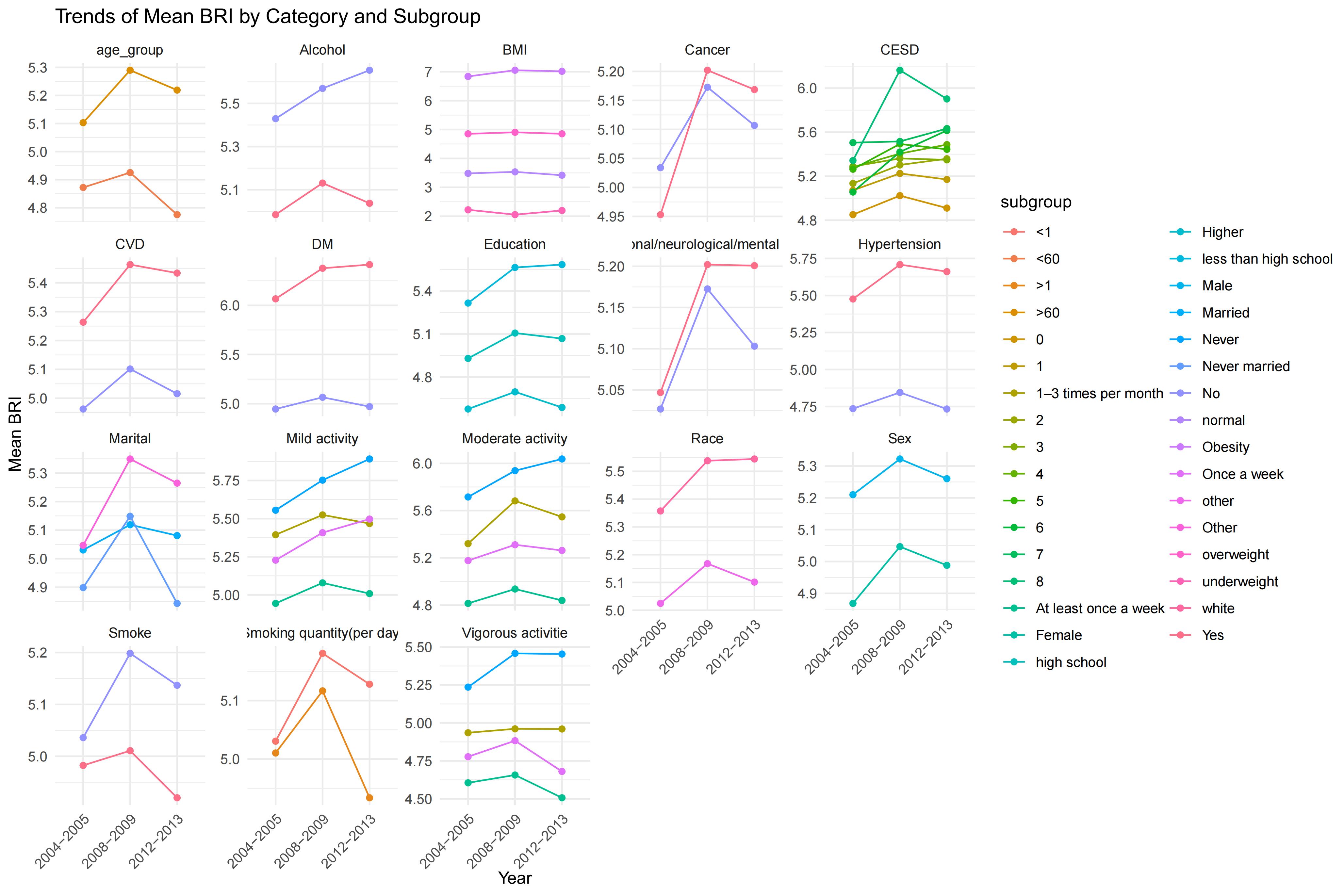  **Supplementary Figure 2** Trends in BRI by Socioeconomic Factors Among ELSA Participants Aged 45 and Older from 2006 to 2019.  *BRI, body roundness index.* |
| --- |

| 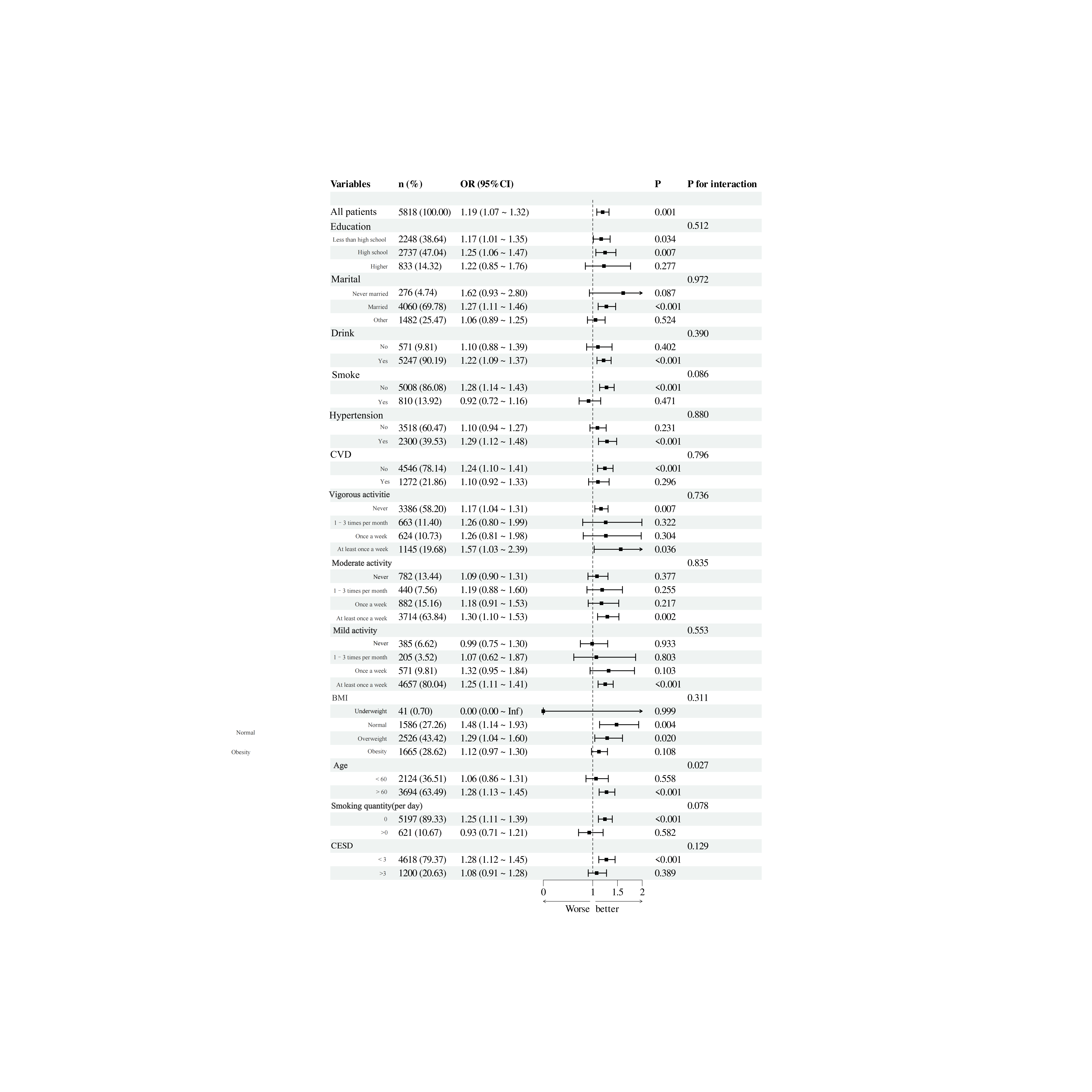 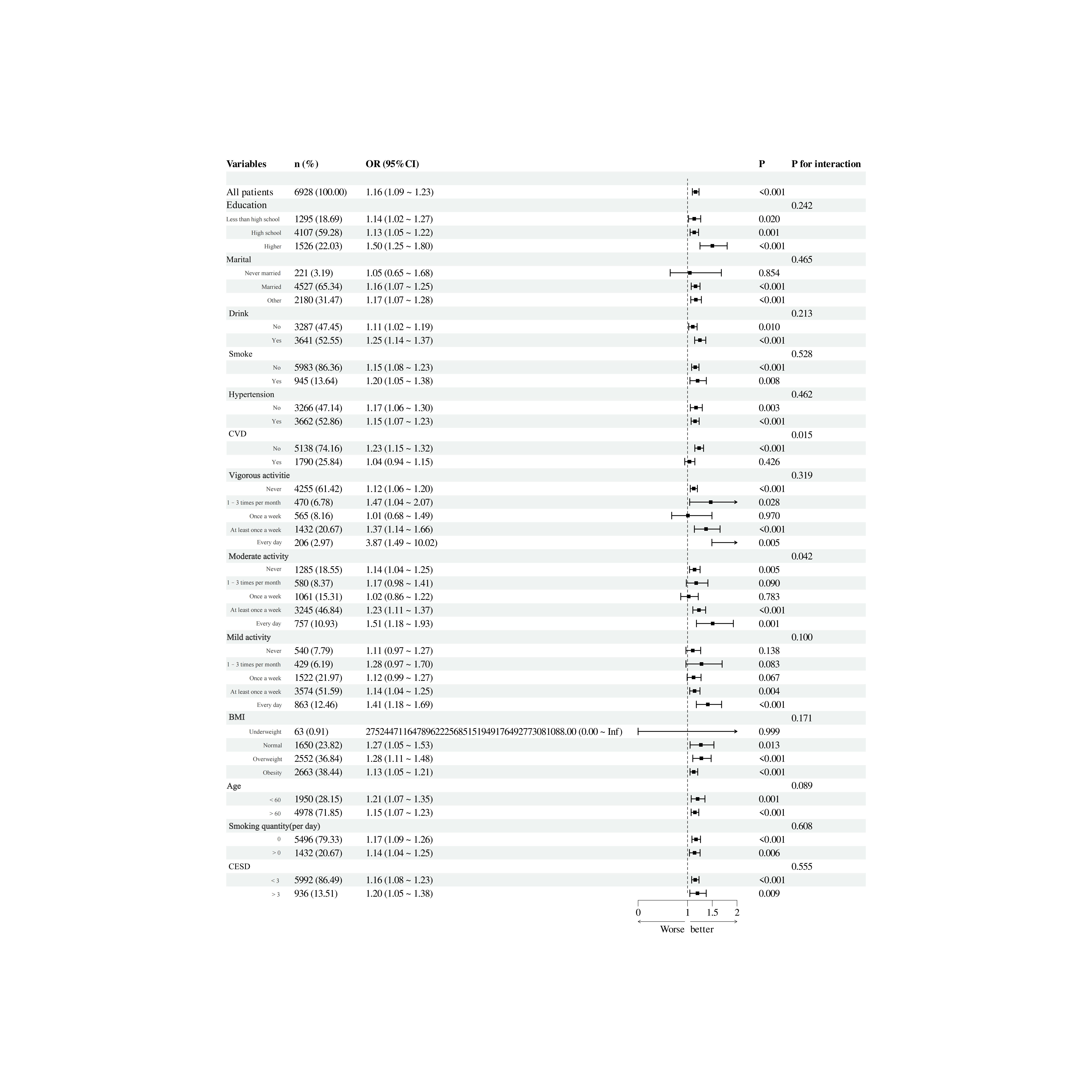  **(A) (B)**  **Supplementary Figure 3** Subgroup analysis of BRI and COPD  *Figure (A) represents Elsa data, and Figure (B) represents HRS data* |
| --- |

| 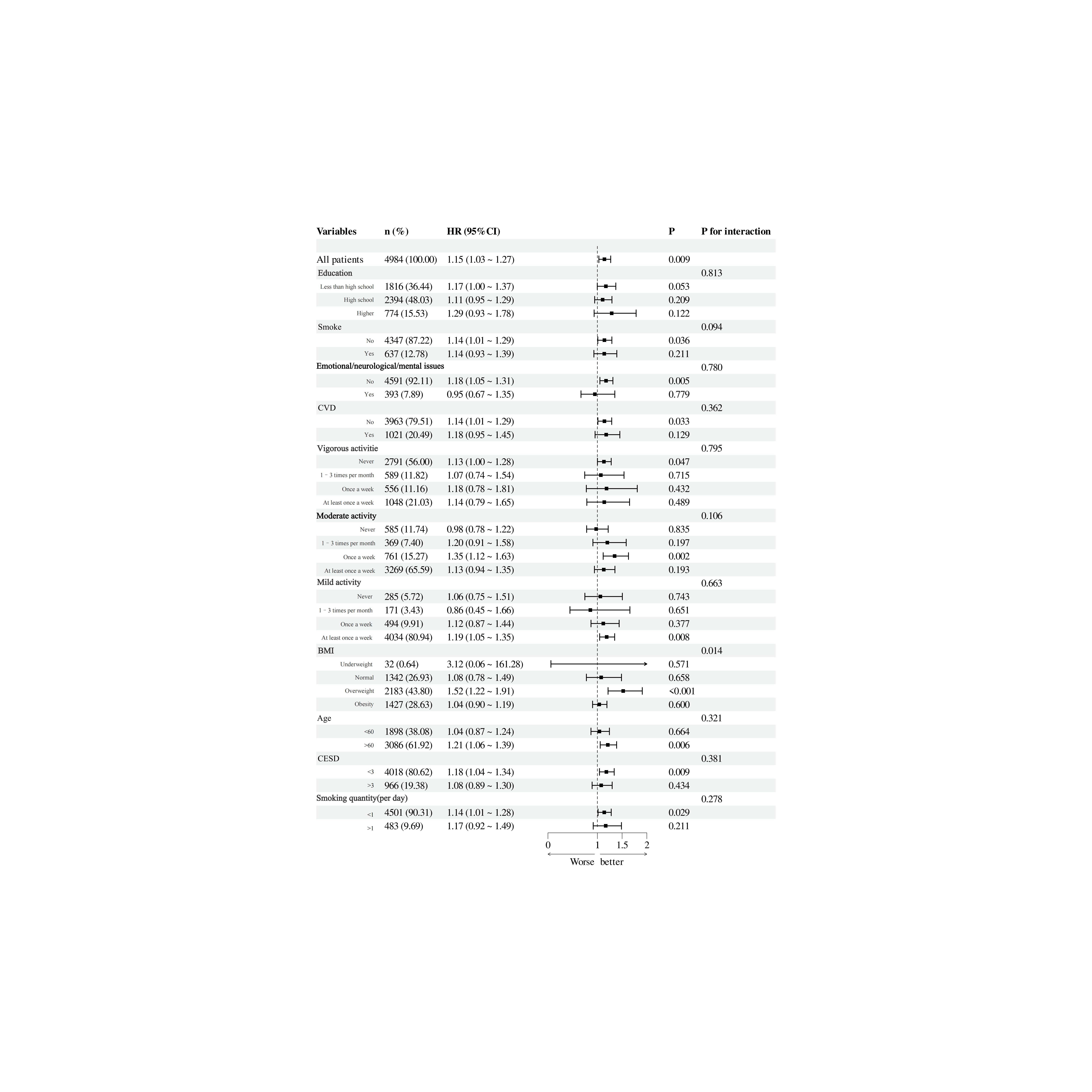  **Supplementary Figure 4** Subgroup Analysis of BRI and Newly Diagnosed COPD in the HRS. |
| --- |

| 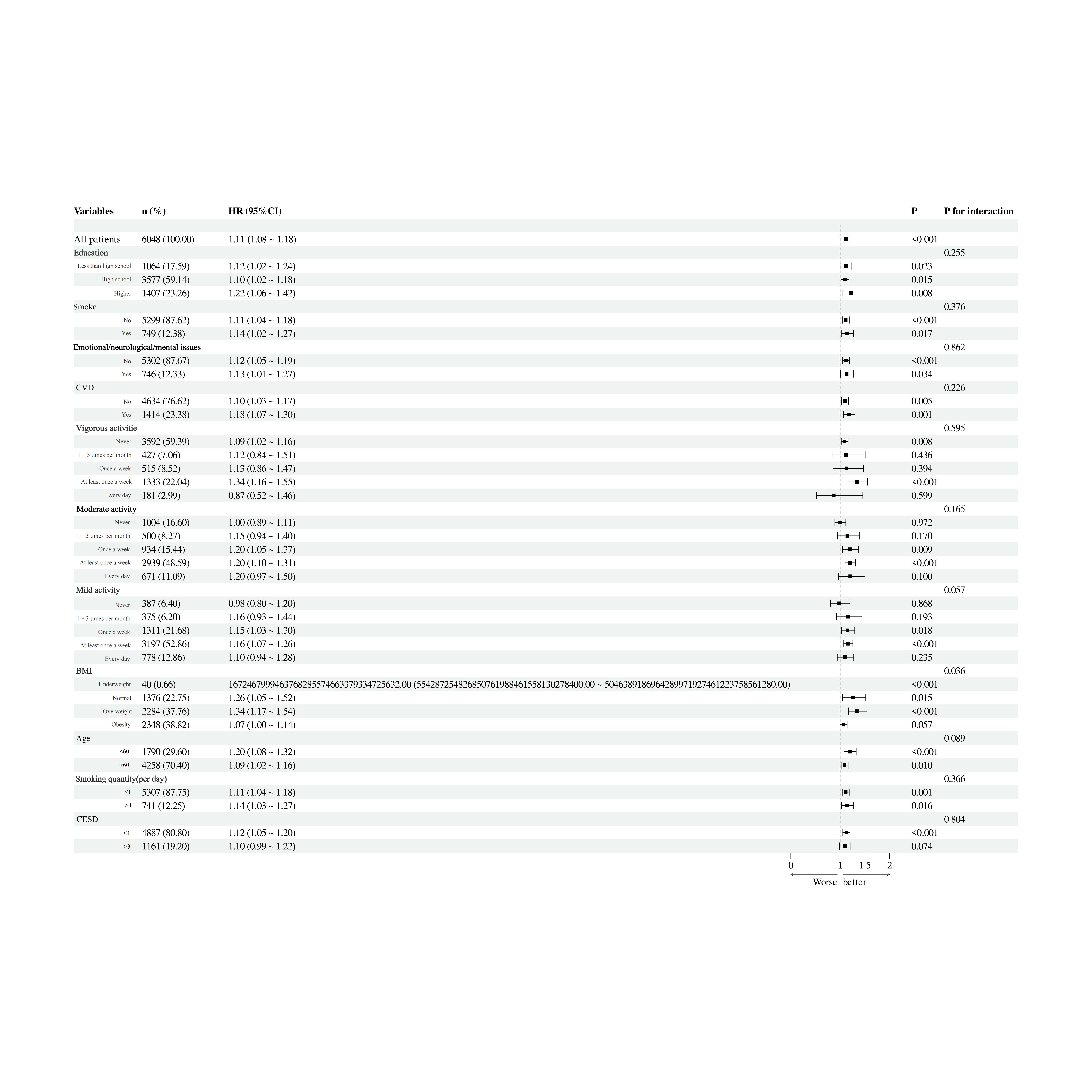  **Supplementary Figure 5** Subgroup Analysis of BRI and Newly Diagnosed COPD in the ELSA. |
| --- |

| 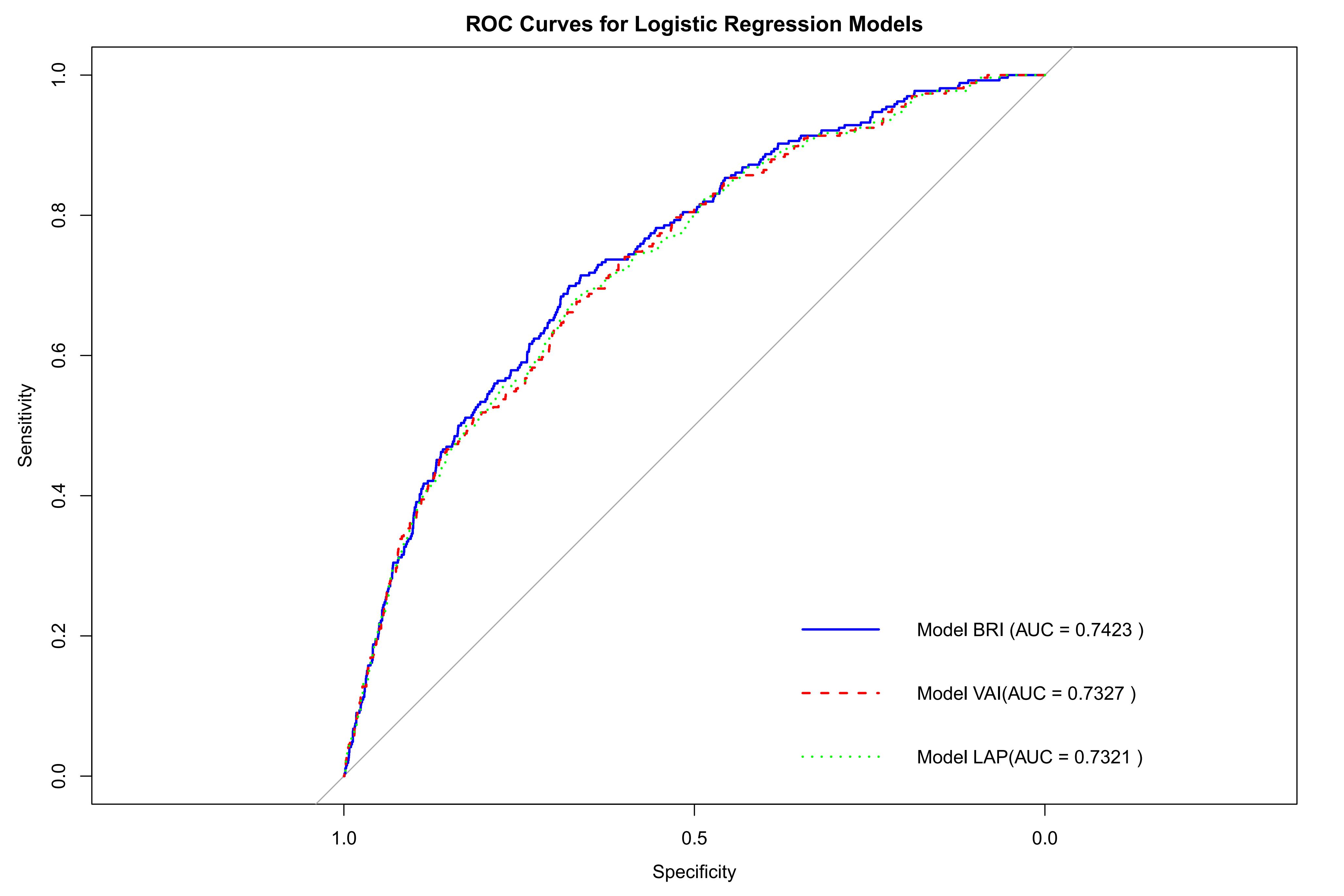  **Supplementary Figure 6** The ROC curve of BRI,LAP and VAI |
| --- |

| **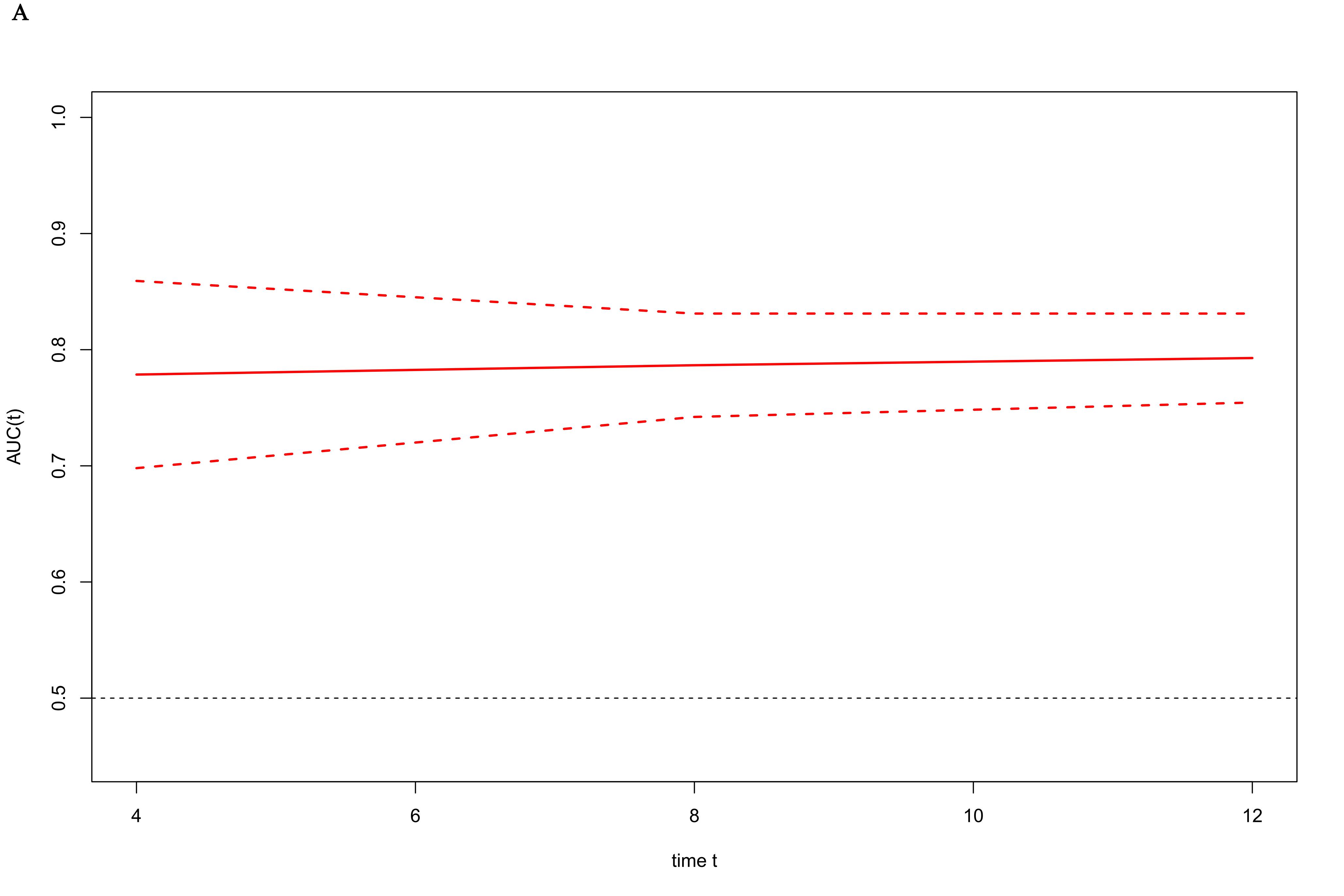**  **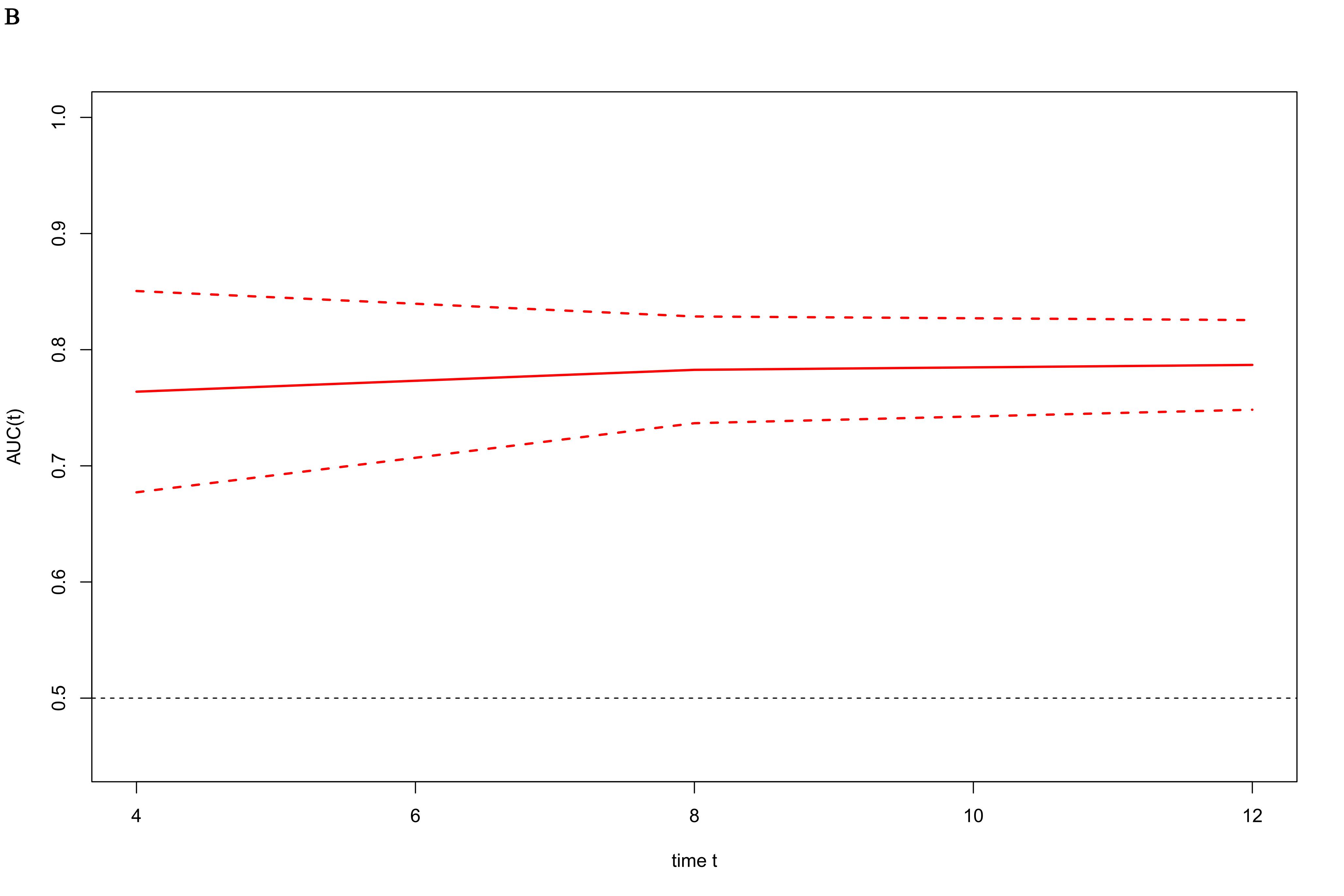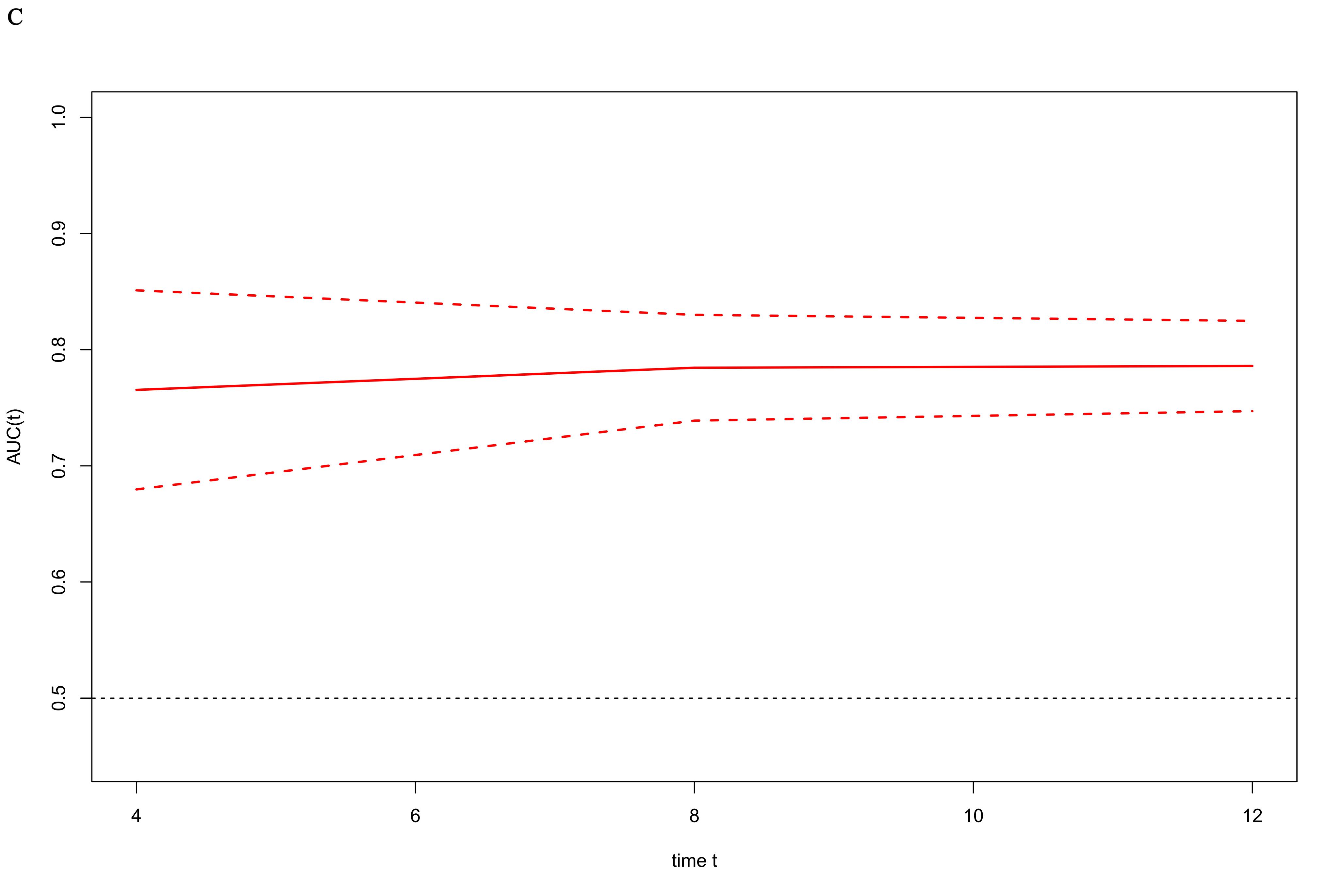**  **Supplementary Figure 7** Time-dependent ROC curve  *(A) Time-dependent ROC curve of BRI for predicting COPD. (B) Time-dependent ROC curve of LAP for predicting COPD. (C) Time-dependent ROC curve of VAI for predicting COPD.* |
| --- |
